# VAPB-mediated ER-targeting stabilizes IRS-1 signalosomes to regulate insulin/IGF signaling

**DOI:** 10.1038/s41421-023-00576-6

**Published:** 2023-08-01

**Authors:** Xiu Kui Gao, Zu Kang Sheng, Ye Hong Lu, Yu Ting Sun, Xi Sheng Rao, Lin Jing Shi, Xiao Xia Cong, Xiao Chen, Hao Bo Wu, Man Huang, Qiang Zheng, Jian-sheng Guo, Liang Jun Jiang, Li Ling Zheng, Yi Ting Zhou

**Affiliations:** 1 Department of Biochemistry and Department of Orthopaedic Surgery of the Second Affiliated Hospital, Zhejiang University School of Medicine, Hangzhou, Zhejiang, China; 2 International Institutes of Medicine, the Fourth Affiliated Hospital of Zhejiang University School of Medicine, Yiwu, Zhejiang China; 3 Dr. Li Dak Sum & Yip Yio Chin Center for Stem Cell and Regenerative Medicine, Zhejiang Provincial Key Lab for Tissue Engineering and Regenerative Medicine, Zhejiang University School of Medicine, Hangzhou, Zhejiang China; 4 Department of Biochemistry and Department of General Intensive Care Unit of the Second Affiliated Hospital, Zhejiang University School of Medicine, Hangzhou, Zhejinag China; 5 Key Laboratory of Multiple Organ Failure (Zhejiang University), Ministry of Education, Hangzhou, Zhejiang China; 6 Department of Pathology of Sir Run Run Shaw Hospital, Center of Cryo-Electron Microscopy, Zhejiang University School of Medicine, Hangzhou, Zhejiang China; 7 ZJU-UoE Institute, Zhejiang University School of Medicine, Hangzhou, Zhejiang China; 8 Cancer Center, Zhejiang University, Hangzhou, Zhejiang China; 9 Liangzhu Laboratory, Hangzhou, Zhejiang China

**Keywords:** Endoplasmic reticulum, Insulin signalling

## Abstract

The scaffold protein IRS-1 is an essential node in insulin/IGF signaling. It has long been recognized that the stability of IRS-1 is dependent on its endomembrane targeting. However, how IRS-1 targets the intracellular membrane, and what type of intracellular membrane is actually targeted, remains poorly understood. Here, we found that the phase separation-mediated IRS-1 puncta attached to endoplasmic reticulum (ER). VAPB, an ER-anchored protein that mediates tethers between ER and membranes of other organelles, was identified as a direct interacting partner of IRS-1. VAPB mainly binds active IRS-1 because IGF-1 enhanced the VAPB-IRS-1 association and replacing of the nine tyrosine residues of YXXM motifs disrupted the VAPB-IRS-1 association. We further delineated that the Y745 and Y746 residues in the FFAT-like motif of IRS-1 mediated the association with VAPB. Notably, VAPB targeted IRS-1 to the ER and subsequently maintained its stability. Consistently, ablation of VAPB in mice led to downregulation of IRS-1, suppression of insulin signaling, and glucose intolerance. The amyotrophic lateral sclerosis (ALS)-derived VAPB P56S mutant also impaired IRS-1 stability by interfering with the ER-tethering of IRS-1. Our findings thus revealed a previously unappreciated condensate-membrane contact (CMC), by which VAPB stabilizes the membraneless IRS-1 signalosome through targeting it to ER membrane.

## Introduction

As the most abundant membrane compartment in eukaryotic cells, the endoplasmic reticulum (ER) forms multiple contacts with other organelles, providing platforms for organelle communication, lipid transfer, and calcium signaling^1–4^. Growing evidence indicates that ER also associates with membraneless organelles, which are formed by biomolecular condensation/phase separation^5–7^. Very recent studies demonstrated that ER membrane surfaces directly influence assembly and size of tethered condensates^5–9^. The VAPs (VAPA and VAPB) are the major players that generate tethers between the ER and the membranes of other organelles^4,10,11^. However, whether VAPs are involved in mediating condensate-membrane contact (CMC) remains largely unexplored.

Insulin/insulin-like growth factor (IGF) signaling modulates diverse pivotal physiological events including differentiation, metabolism, and growth^12–22^. Acting as a critical node in insulin/IGF pathway, the scaffold proteins insulin receptor substrate 1 (IRS-1) recruits and activates downstream Src homology 2 (SH2)-containing effectors^23–25^. It was reported that IRS-1 displays puncta structures in cells^26–28^, which has recently been identified as an outcome of self-association-mediated condensation/phase separation by our group^29^. It has long been recognized that the intracellular membrane attachment of IRS-1 correlates to its phosphorylation status and determines its turnover^23,30–35^. The intracellular membrane fraction harbors Tyr-phosphorylated active IRS-1, while cytosolic redistribution of IRS-1 leads to degradation^31–35^. However, what type of intracellular membrane IRS-1 is actually targeted is poorly understood. Given that there is no transmembrane domain in IRS-1, how IRS-1 signalosomes target the intracellular membrane also remains unknown.

In this study, we found that the phase separation-mediated IRS-1 droplets target to the ER. Our screening further identified that the ER-anchored protein VAPB is one of the potential interaction partners of IRS-1. Indeed, we delineated the critical amino acid residues mediating the IRS-1–VAPB interaction and showed that the CMC mediated by VAPB stabilizes IRS-1 by targeting it to the ER. Knockout of VAPB led to reduced IRS-1 expression, impaired insulin signaling, and aberrant glucose homeostasis in mice. Consistently, the amyotrophic lateral sclerosis (ALS)-derived VAPB P56S mutant impairs stability and ER-tethering of IRS-1. Based on these observations, we concluded that the ER, and its VAPB-mediated tethers to membraneless IRS-1 signalosomes, could be linked to the regulation of insulin/IGF signaling.

## Results

### Phase separation-mediated IRS-1 droplets target to the ER

**Fig. 1:**
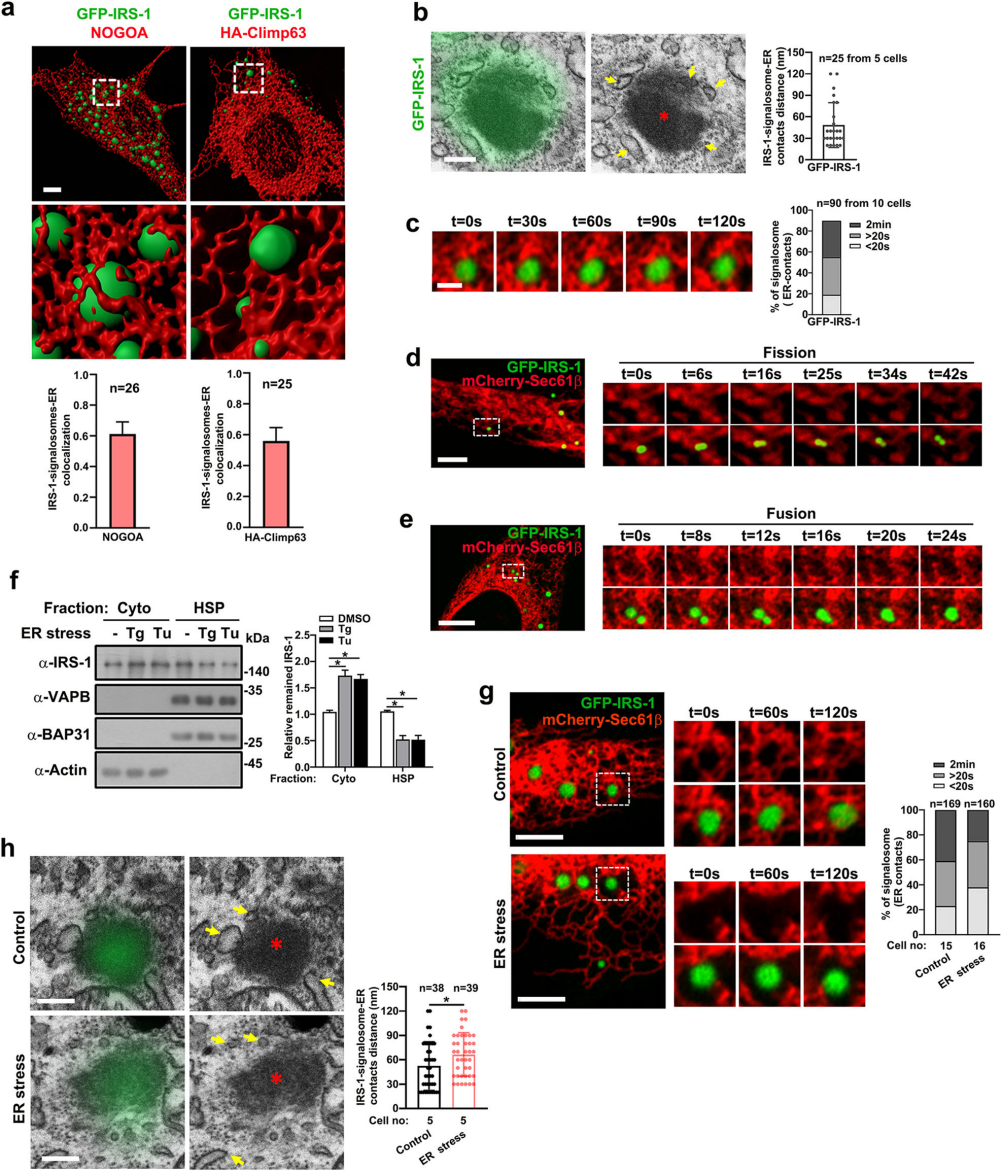
The membraneless IRS-1 condensates are associated with ER. **a** 3D rendering of Z-stacking confocal images of representative GFP-IRS-1 C2C12 myoblasts stained with endogenous NOGOA (left panel) or HA-Climp63 (right panel). Scale bar, 5 µm. **b** Correlative light and electron microscopy (CLEM) of C2C12 cells transiently transfected with GFP-IRS-1. Scale bar, 0.5 µm. A red asterisk (*) indicates a nonmembrane GFP-IRS-1 foci while yellow arrows indicate ER. Minimum distance of ER located from the considered IRS-1 foci was quantified (*n* = 25 from 5 cells). Data are represented as mean ± SD. **c** Representative live imaging of GFP-IRS-1 droplet tethered with ER membrane for > 2 min. Quantification of ER contact of IRS-1 puncta from ten cells (*n* = 90 IRS-1 puncta from 10 cells). Scale bar, 1 µm. **d** A representative merged image of the ER (red) labeled with mCherry-Sec61β and GFP-IRS-1 (green) in C2C12 cells. Scale bar, 5 µm. Insets are time-lapse images showing the fission of a GFP-IRS-1 granule contacts with the ER. **e** A representative merged image of the ER and GFP-IRS-1 and insets showing the fusion of two GFP-IRS-1 granules contact with the ER. Scale bar, 5 µm. **f** C2C12 myoblasts treated with DMSO, Tu (2 μg/mL), or Tg (1 μM) for 3 h were homogenized and extracts were fractionated into cytosol and HSP (high-speed pellet). Fractions were analyzed by western blotting with 20 µg of protein loaded in each lane. Data in the bar graphs represent the mean ± SEM values of the ratios of densities for three independent experiments. *P < 0.05. **g** Representative live imaging of GFP-IRS-1 droplets and ER membrane in DMSO- or Tg-treated C2C12 cells. Cells were quantified for the degree of the ER-tethering of IRS-1 puncta. Scale bars, 5 µm. **h** CLEM of DMSO- or Tg-treated C2C12 cells expressing GFP-IRS-1. Scale bar, 0.5 µm. Red asterisks (*) indicate nonmembrane GFP-IRS-1 foci while yellow arrows indicate ER. Minimum distance of ER located from the considered IRS-1 foci was quantified. Data are represented as mean ± SD. *P < 0.05.

We recently revealed that IRS-1 undergoes phase separation to form insulin/IGF signalosomes^29^. The localization of IRS-1 to the intracellular membrane compartments has long been recognized as pivotal for the determination of its activation and stability^31–34^. It is of interest to determine the type of intracellular membrane that IRS-1 droplets target to. By co-transfecting IRS-1 and a series of organelle markers, we found that IRS-1 droplets were surrounded by both Climp63- and NOGOA-positive ER structures (Fig. 1a; Supplementary Fig. S1a), and were also occasionally attached to early endosomes, as previously recognized^36^, and mitochondria (Supplementary Fig. S1b). These observations are consistent with a previous study showing that IRS-1 localize to an ER-enriched fraction in β-cells^37^. We further performed electron microscopy (EM) coupled with correlative confocal imaging analysis (Supplementary Fig. S1c), which revealed that the membraneless IRS-1 spheres were surrounded by ER structures (Fig. 1b). We also quantified the minimum distance of ER located from the IRS-1 foci and found that a substantial portion was < 30 nm (Fig. 1b).

Previous studies measured the contact time between ER and membrane bound or membraneless organelles to validate their tethering^38,39^. This is because only sustained contacts between condensates and ER tubules over time indicate the tethering between the two organelles^38,39^. As previously described^38,39^, we used live-cell imaging to measure the extent to which IRS-1 droplets remained tethered to the ER over time. About 40% of IRS-1 puncta stably associated with the ER throughout a 2-min live-cell imaging (Fig. 1c), verifying that a substantial subset of IRS-1 puncta is tethered to the ER. Moreover, IRS-1 granules contacted to ER underwent both fission and fusion, suggesting the liquid nature of ER-tethered IRS-1 puncta (Fig. 1d, e). These results thus revealed a novel type of condensate-membrane contact.

Cellular IRS-1 exists mainly in two subcellular fractions: the high-speed pellet (HSP) fraction (also designated as the low-density membrane fraction) and the cytosolic fraction^31–35,40^. We further examined if disruption of ER function, via initiation of ER stress, affects the membrane targeting of IRS-1. Both tunicamycin (Tu) and thapsigargin (Tg) treatments evoked ER stress by inhibiting protein N-glycosylation and ER Ca^2+^ ATPase, respectively^41^ (Supplementary Fig. S2a). As previously described^31–35,40^, we prepared fractionations from control and Tg- or Tu-treated cellular extracts to examine the levels of IRS-1 in the HSP and soluble fractions. In the HSP fractions, compared with control cells, the levels of endogenous IRS-1 were obviously downregulated (Fig. 1f). Indeed, contact time measurement and confocal microscopy analysis revealed that Tg treatment promoted the ER detachment of GFP-IRS-1 droplets (Fig. 1g; Supplementary Fig. S2b). Correlative light and electron microscopy (CLEM) also showed reduced tethering between IRS-1 condensates and ER (Fig. 1h). A very recent study revealed that the condensates formed on ER membrane are less mobile^8^. We thus performed fluorescence recovery after photobleaching (FRAP) analysis to measure the recovery half time of GFP-IRS-1, which provides insights into how fast the labeled molecules are diffusing. Consistently, the IRS-1 puncta attached to ER displayed slower recovery rates (Supplementary Fig. S3), indicating a less liquid-like behavior. We thus concluded that ER stress leads to detachment of IRS-1 droplets from endomembrane, which is most likely ER membrane. Together, all these findings indicated that phase separation-mediated IRS-1 puncta are tethered to the ER.

### IRS-1 directly interacts with ER-anchored molecule VAPB

**Fig. 2:**
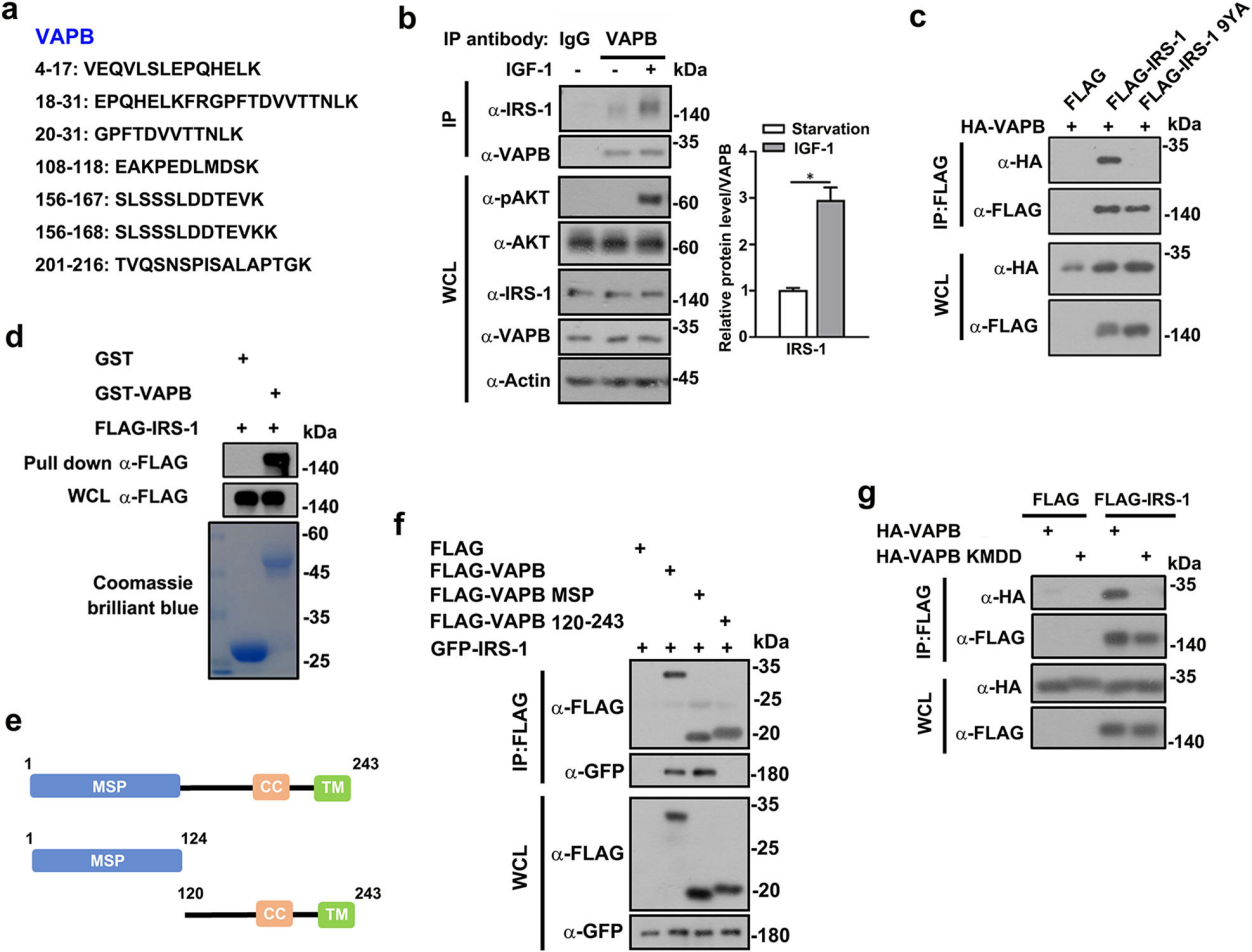
VAPB directly interacts with IRS-1. **a** The gel lane was subjected to trypsin digestion followed by MALDI-TOF analysis, and VAPB was identified as a potential interacting partner of IRS-1. Peptides identified by MS analysis of FLAG-IRS-1 immunoprecipitates were listed. **b** C2C12 myoblasts, treated with or without 100 ng/mL IGF-1 for 2.5 min after serum starvation for 16 h, were subjected to immunoprecipitation with IRS-1 antibodies. Coimmunoprecipitated IRS-1 and VAPB were detected by Western blot analysis. The relative IRS-1 levels co-precipitated by VAPB were quantified. *P < 0.05. **c** FLAG-tagged IRS-1 or 9YA mutant was co-transfected with HA-VAPB into 293 T cells for co-IP analysis. **d** GST or GST-VAPB fusion protein was incubated with purified FLAG-tagged IRS-1 for direct Pull-down assay. **e** Schematic diagram of VAPB and its mutants. **f** FLAG-tagged IRS-1 or mutant constructs as shown in (**e**) were co-transfected with GFP-IRS-1 into 293 T cells for co-IP assays. **g** FLAG-tagged IRS-1 was co-transfected with HA-VAPB or VAPB KMDD mutant into 293 T cells for co-IP analysis.

We next set out to explore the mechanism by which IRS-1 attached to ER. To this aim, we performed a proteomics screening by co-immunoprecipitating FLAG-IRS-1 to identify interaction partners. The gel lanes were excised and subjected to mass spectrometry (MS) analysis, which revealed that the ER-anchored molecule VAPB is a potential candidate interacting partner of IRS-1 (Fig. 2a). VAPB, together with VAPA, generates tethers between the membranes of ER and other organelles^42^. However, the roles of VAPB in mediating ER and membraneless condensates remains largely unexplored. We thus hypothesized that the IRS-1 puncta might attach to ER through interacting with VAPB. To test this postulation, we first validated the VAPB–IRS-1 interaction by immunoprecipitating endogenous VAPB. Endogenous IRS-1 were coimmunoprecipitated with VAPB and this interaction was enhanced by IGF-1 stimulation (Fig. 2b), suggesting the involvement of VAPB–IRS-1 complex in insulin/IGF signaling. The endogenous interaction between VAPB and IRS-1 was impaired by Tg- or Tu-induced ER stress (Supplementary Fig. S4). Consistently, an IRS-1 9YA construct, where the nine tyrosine residues of YXXM motifs were mutated to alanine^29^, failed to interact with VAPB (Fig. 2c), indicating that VAPB only binds active IRS-1.

We further purified recombinant FLAG-tagged IRS-1 for direct pull-down analysis using GST and GST-VAPB prepared in *Escherichia coli*. We found that IRS-1 directly bound VAPB (Fig. 2d). VAPB interacts with numerous partners through its MSP domain to mediate ER-organelle tethering^11,43–45^. The co-immunoprecipitation (Co-IP) assay demonstrated that the MSP domain of VAPB mediated its association with IRS-1 (Fig. 2e, f). The MSP domain of VAPB recognizes FFAT-like (two phenylalanines [FF] in an acidic tract) motifs to mediate the interaction between VAPB and its binding partners^42^. In line with this, Co-IP assays demonstrated that the FFAT motif-binding-deficient mutant of VAPB (K87D and M89D, herein referred to as VAPB KMDD mutant) failed to associate with IRS-1 (Fig. 2g). Consistently, VAPB KMDD mutant displayed impaired interaction ability with FFAT motif-containing proteins including ULK1 and STARD3 (Supplementary Fig. S5). These results indicate that IRS-1 directly interacts with ER-anchored molecule VAPB.

### An FFAT-motif in IRS-1 mediates the association with VAPB

**Fig. 3:**
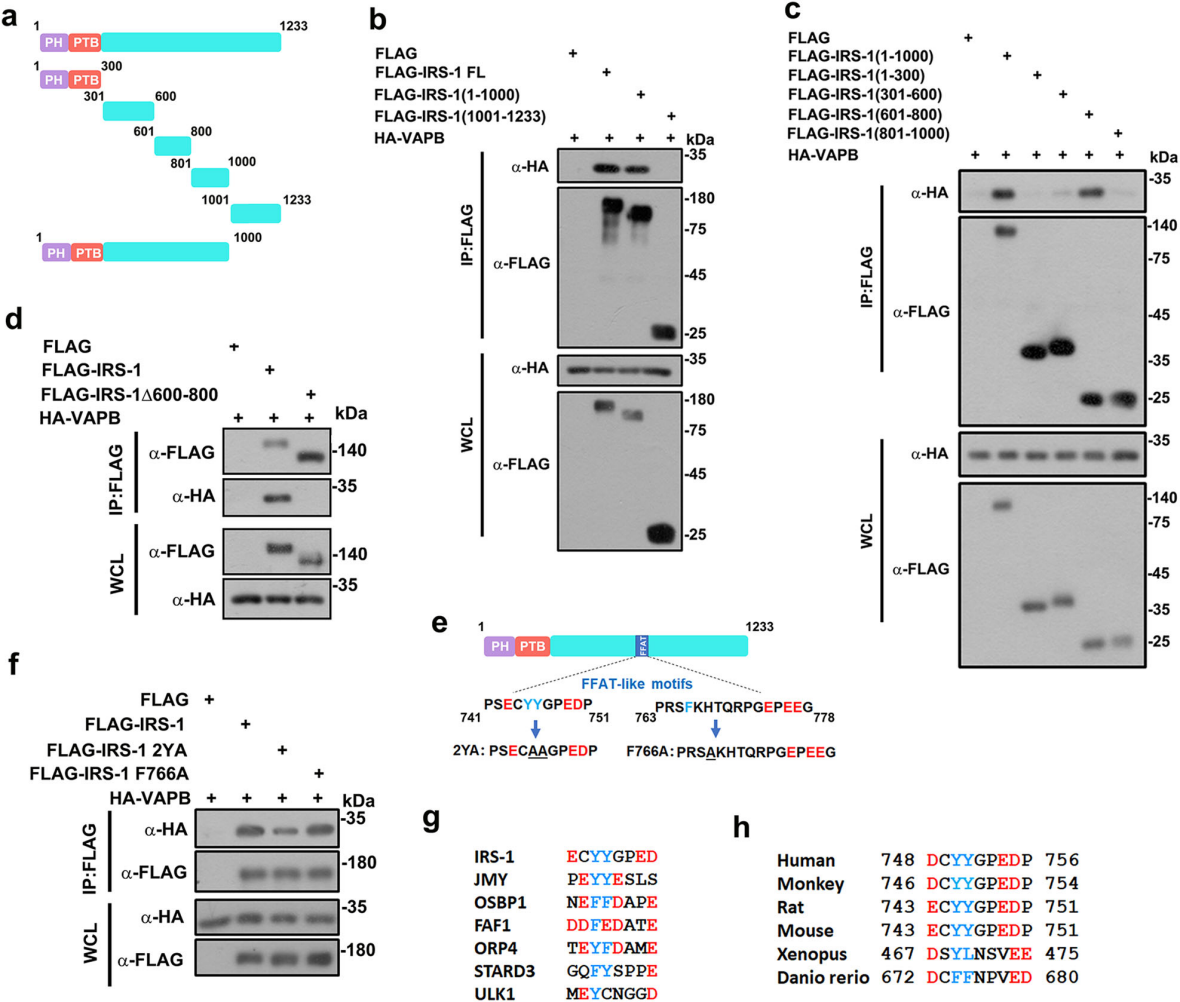
The FFAT motif in IRS-1 mediates the association with VAPB. **a** Schematic diagram of IRS-1 and its truncation mutants. **b**, **c** Co-IP assay between HA-VAPB and the IRS-1 mutants as shown in **a**. **d** FLAG-tagged IRS-1 or Δ600–800 mutant constructs were co-transfected with HA-VAPB into 293 T cells for co-IP assays. **e** Two putative FFAT motives were identified in the mouse IRS-1 sequence. The Y745 Y746 or F766 residue of the two motifs were replaced by alanine residues. **f** FLAG-tagged IRS-1, 2YA, or F766A mutant was co-transfected with HA-VAPB into 293 T cells for co-immunoprecipitation assays. **g** Alignment of IRS-1 FFAT motif with FFAT motifs of known VAPB interacting partners. **h** Conservation of IRS-1 FFAT motifs.

We next created a series of IRS-1 truncation mutants, as shown in Fig. 3a, to identify the VAPB-binding region on IRS-1. The co-IP assay indicated that amino acids 601–800 of IRS-1 were required for its association with VAPB (Fig. 3b, c). We further prepared a Δ600–800 mutant and validated the requirement of this region for interacting with VAPB (Fig. 3d). Two potential FFAT-like motifs were identified within the 601–800 region of IRS-1 (Fig. 3e). We therefore created two IRS-1 mutant constructs in which a double (Y745, Y746; herein referred to as IRS-1-2YA) tyrosine residues or a single (F766) phenylalanine residue were changed to alanine. Binding of IRS-1 to VAPB was abolished by the Y745 Y746 double mutation, but not the F766 single mutation (Fig. 3f). The FFAT motif of IRS-1 has a high degree of similarity to those of other known VAPB binding partners (Fig. 3g; Supplementary Fig. S5). It is conserved among mammalian species (Fig. 3h). We thus identified the FFAT motif in IRS-1 that mediates the association with VAPB.

### IRS-1 droplets target to ER in a VAPB-dependent manner

**Fig. 4:**
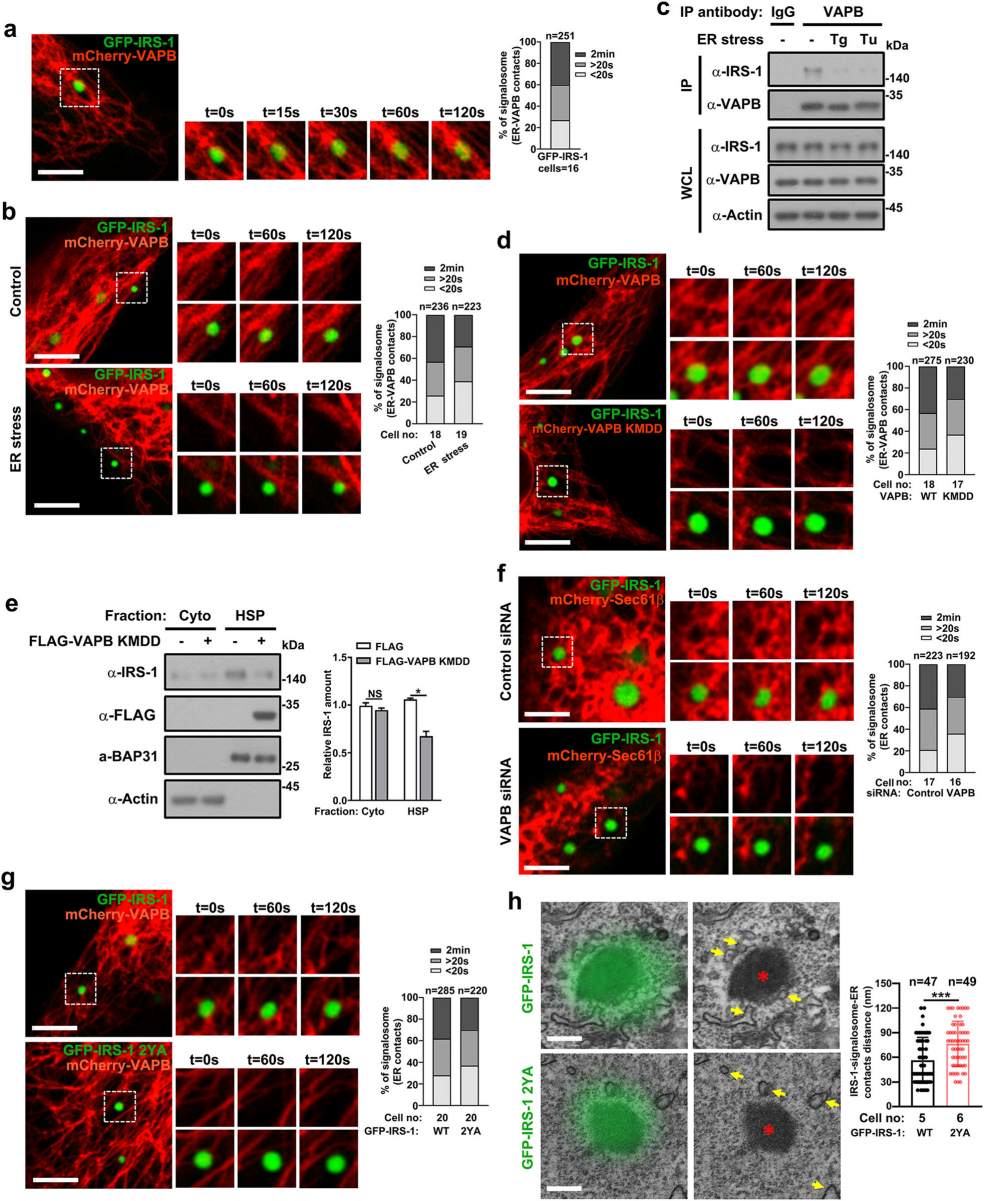
VAPB mediates the ER-targeting of IRS-1 droplets. **a** Representative live imaging of GFP-IRS-1 droplets and mCherry-VAPB in C2C12 cells. Cells were quantified for the degree of association between VAPB and IRS-1 signalosomes. Scale bar, 4 µm. **b** Representative live imaging of GFP-IRS-1 droplets and mCherry-VAPB in DMSO- or Tg-treated C2C12 cells. Cells were quantified for the degree of association between VAPB and IRS-1 signalosomes. Scale bar, 4 µm. **c** C2C12 myoblasts, treated with Tg or Tu for 3 h, were subjected to immunoprecipitation with VAPB antibodies. Coimmunoprecipitated IRS-1 and VAPB were detected by Western blot analysis. **d** Representative live imaging of GFP-IRS-1 droplets and mCherry-VAPB or mCherry-VAPB KMDD mutant in C2C12 cells. Cells were quantified for the degree of association between IRS-1 signalosomes and VAPB wildtype or KMDD mutant. Scale bars, 4 µm. **e** C2C12 myoblasts transfected with FLAG-tagged control or VAPB KMDD plasmids for 24 h were homogenized and extracts were fractionated into cytosol and HSP. Fractions were analyzed by western blotting and quantified. Data in the bar graphs represent the mean ± SEM values of the ratios of densities for three independent experiments. *P < 0.05. **f** Representative live imaging of GFP-IRS-1 droplets and mCherry-Sec61β in C2C12 cells transfected with control or VAPB siRNA. Cells were quantified for the degree of association between IRS-1 signalosomes and ER. Scale bars, 4 µm. **g** Representative live imaging of GFP-IRS-1 or GFP-IRS-1 2YA mutant droplets and mCherry-VAPB in C2C12 cells. Cells were quantified for the degree of association between IRS-1 signalosomes and VAPB. Scale bars, 4 µm. **h** CLEM of C2C12 cells expressing GFP-IRS-1 or GFP-IRS-1 2YA mutant. Scale bar, 0.4 µm. Red asterisks (*) indicate nonmembrane GFP-IRS-1 foci while yellow arrows indicate ER. Minimum distance of ER located from the considered IRS-1 foci was quantified. Data are represented as mean ± SD. ***P < 0.001.

To examine if IRS-1-mediated insulin/IGF signalosome attached to ER through interacting with VAPB, we first examined the tethering of VAPB and IRS-1 droplets. IRS-1 has been shown to display puncta structures in cells^26,27^ and our group recently revealed that the C-terminus of IRS-1 undergoes phase separation to mediate the formation of insulin/IGF signalosomes^29^. VAPB displayed typical ER-structure (Supplementary Fig. S6a, b), as previously described^44^. Sustained contacts between GFP-IRS-1 condensates and VAPB tubules were observed, indicating the tethering between the two structures (Fig. 4a). In contrast, Tg treatment impaired the tethering of VAPB and IRS-1 signalosomes (Fig. 4b; Supplementary Fig. S7a). Consistently, the VAPB–IRS-1 interaction was impaired upon Tg or Tu treatment (Fig. 4c).

Moreover, the VAPB KMDD mutant also displayed reduced tethering with IRS-1 foci (Fig. 4d; Supplementary Fig. S7b) and reduced the IRS-1 levels in the HSP fraction (Fig. 4e). Similarly, knockdown of VAPB impaired the tethering between IRS-1 droplets and ER (Fig. 4f; Supplementary Fig. S7c). These results indicate that IRS-1 droplets target to the ER through interacting with VAPB. This was further supported by the finding that the IRS-1 2YA mutant, which loss interaction capability with VAPB (Fig. 3f), displayed an impaired colocalization with VAPB and ER (Fig. 4g, h; Supplementary Fig. S7d). We thus concluded that IRS-1 granules tethered to ER in a VAPB-dependent manner.

### VAPB stabilizes IRS-1 to regulate insulin/IGF signaling

**Fig. 5:**
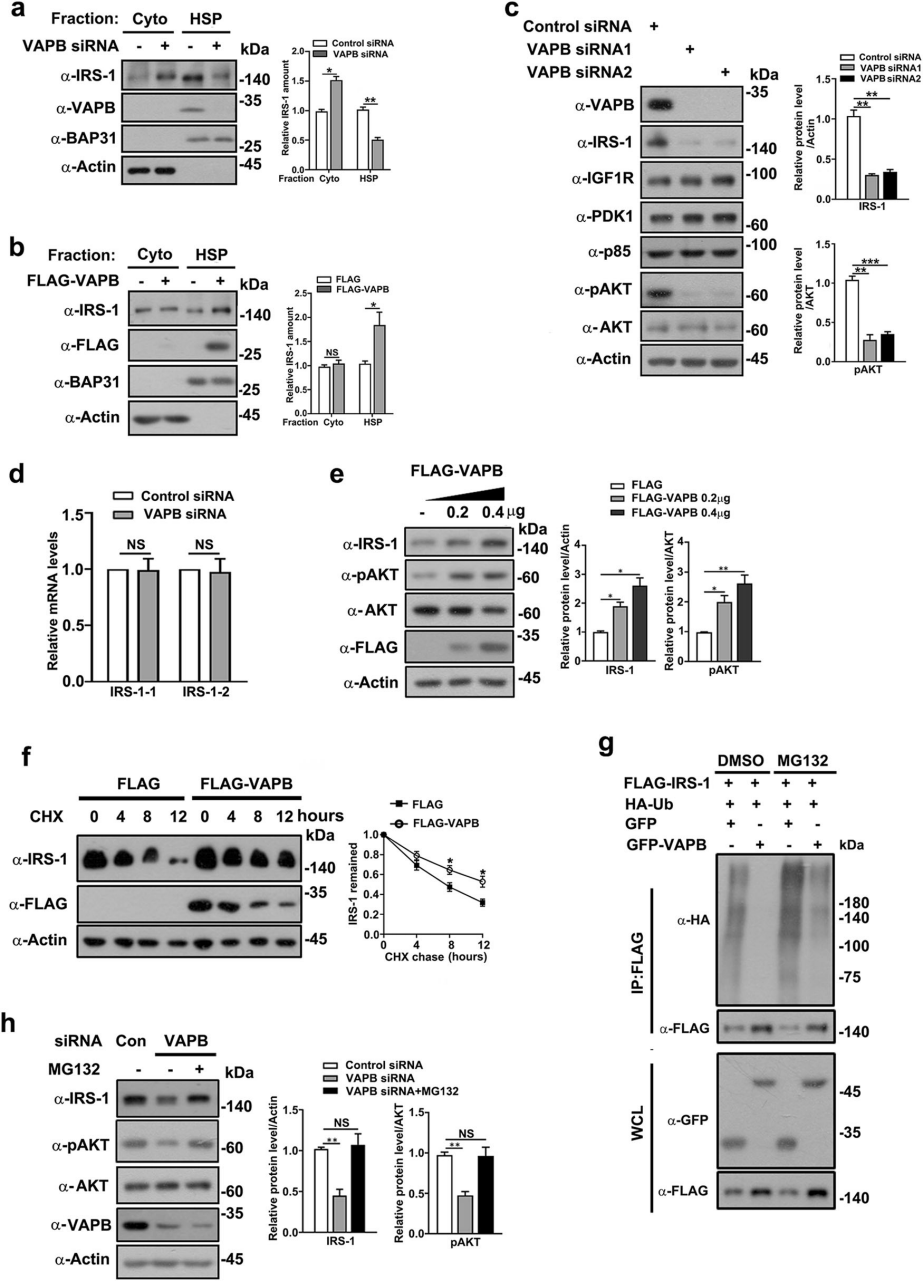
VAPB stabilizes IRS-1. **a** C2C12 myoblasts transfected with control or VAPB siRNA for 24 h were homogenized and extracts were fractionated into cytosol and HSP. Fractions were analyzed by western blotting and quantified. Data in the bar graphs represent the mean ± SEM values of the ratios of densities for three independent experiments. *P < 0.05, **P < 0.01. **b** C2C12 myoblasts stably expressing control FLAG vector or FLAG-VAPB were processed as in **a**. Fractions were analyzed by western blotting and quantified. Data in the bar graphs represent the mean ± SEM values of the ratios of densities for three independent experiments. *P < 0.05, ns: not significant. **c** Lysates of C2C12 myoblasts transfected with either control or two VAPB siRNAs for 48 h were western blotted with the indicated antibodies of IGF signaling proteins. Data in the bar graphs represent the mean ± SEM values of the ratios of densities for three independent experiments. **P < 0.01. ***P < 0.001. **d** C2C12 cells transfected with control or VAPB siRNA for 48 h were subjected to qRT-PCR for transcription levels of *IRS-1*. Data in the bar graphs represent the mean ± SEM values. ns: not significant. **e** Lysates of C2C12 cells transfected with FLAG-VAPB were subjected to Western blot analysis for the indicated antibodies. Data in the bar graphs represent the mean ± SEM values of the ratios of densities for three independent experiments. *P < 0.05. **P < 0.01. **f** C2C12 cells transfected with FLAG vectors or FLAG-VAPB were treated with cycloheximide (CHX) (25 µg/mL) for the indicated times. Cell lysates were subjected to Western blot analysis with the IRS-1 antibody. The expression levels of IRS-1 were quantified (right panel). **g** FLAG-VAPB and HA-ubiquitin were co-transfected with GFP or GFP-VAPB into C2C12 myoblasts with the indicated combination for 36 h. After 5 h of MG132 (20 μM) treatment, IRS-1 ubiquitination was determined by immunoprecipitation with a FLAG antibody and immunoblotted with an HA antibody. **h** C2C12 cells were transfected with control or VAPB siRNA for 36 h followed by incubation with DMSO (control) or the proteasome inhibitor MG132 (20 µM) for 5 h. Cell lysates were subjected to Western blot analysis with the indicated antibodies. Data in the bar graphs represent the mean ± SEM values of the ratios of densities for three independent experiments. **P < 0.01.

Since the intracellular membrane localization is essential for stabilizing IRS-1, we further investigated if VAPB stabilizes IRS-1 through intermediating its ER localization. Knockdown of VAPB significantly reduced the membrane fraction of IRS-1 and elevated cytosolic levels of IRS-1 (Fig. 5a). Conversely, the ectopic expression of VAPB resulted in an increase in membrane-targeted IRS-1 (Fig. 5b). In accordance with the notion that the intracellular membrane targeting of IRS-1 is essential for maintaining its stability^31–36,46^, depletion of VAPB for an extended time period specifically attenuated the expression levels of IRS-1, but not other insulin/IGF signaling molecules including IGFR, PDK-1, and the p85 subunit of PI3K, and subsequently suppressed AKT activation (Fig. 5c; Supplementary Fig. S8a). The membrane fraction verified the reduction of IRS-1 from both HSP and cytoplasm in cells depleted of VAPB for an extended time period (Supplementary Fig. S8b). qPCR analysis showed that IRS-1 transcript levels were not affected (Fig. 5d). In line with this, exogenously expressed VAPB enhanced IRS-1 expression levels (Fig. 5e), delayed the decay of endogenous IRS-1 in myoblasts treated with the translational inhibitor cycloheximide (Fig. 5f), and reduced IRS-1 ubiquitination (Fig. 5g). The proteasome inhibitor MG132 rescued both IRS-1 expression levels and AKT phosphorylation in VAPB-depleted myoblasts (Fig. 5h). Taken together, these results suggest that VAPB regulates IRS-1 turnover by coordinating its ER-membrane targeting.

### Ablation of VAPB downregulates IRS-1 expression and insulin signaling in vivo

**Fig. 6:**
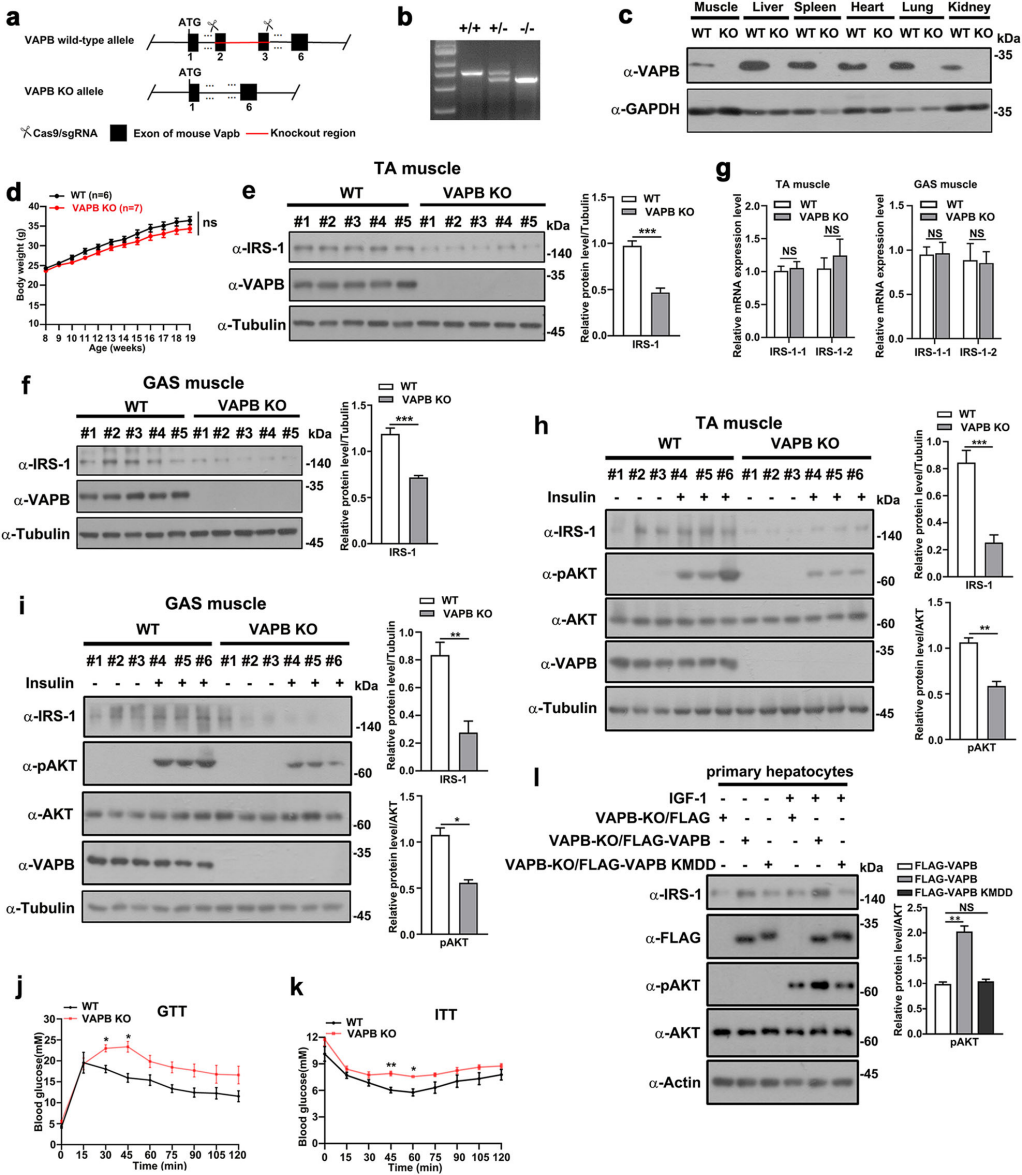
Knockout of VAPB downregulates IRS-1 and insulin signaling in vivo. **a** Gene targeting strategy for *VAPB* knockout. **b** Genomic PCR of WT and VAPB-KO mice. **c** Western blot analyses confirm the ablation of VAPB in various VAPB-KO mouse tissues. **d** Body weight of WT (*n* = 6) and VAPB-KO mice (*n* = 7). ns: not significant. **e** Tibialis anterior (TA) muscles of WT and VAPB-KO mice (*n* = 5 for each group) were harvested for Western blot analysis to determine the expression levels of IRS-1. Data in the bar graphs represent the mean ± SEM values. ***P < 0.001. **f** Gastrocnemius (GAS) muscles of WT and VAPB-KO mice (*n* = 5 for each group) at 11 weeks of age were harvested for Western blot analysis to determine the expression levels of IRS-1. Data in the bar graphs represent the mean ± SEM values. ***P < 0.001. **g** TA and GAS muscles of WT and VAPB-KO mice (*n* = 5 for each group) at 18–20 weeks of age were harvested for qPCR analysis for transcription levels of *IRS-1*. Data in the bar graphs represent the mean ± SEM values. ns: not significant. **h** Phosphorylation of AKT (S473) in TA muscles of the WT and VAPB-KO mice at 9 months of age in response to insulin stimulation. Data in the bar graphs represent the mean ± SEM values. ***P < 0.001, **P < 0.01. **i** Phosphorylation of AKT (S473) in GAS muscles of the WT and VAPB-KO mice at 9 months of age in response to insulin stimulation. Data in the bar graphs represent the mean ± SEM values. *P < 0.05, **P < 0.01. **j** Blood glucose levels during intragastric GTT in male WT and VAPB-KO mice at 18–20 weeks of age. The values show the glucose area under the curve during GTT. WT, *n* = 4; VAPB-KO, *n* = 5. Data are represented as mean ± SEM. *P < 0.05. **k** Blood glucose levels during ITT in male WT and VAPB-KO mice at 18–20 weeks of age. The values show glucose area above the curve during ITT. WT, *n* = 6; VAPB-KO, *n* = 6. Data are represented as mean ± SEM. *P < 0.05, **P < 0.01. **l** Mouse primary hepatocytes isolated from VAPB-KO mice were transfected with FLAG control vector, FLAG-VAPB, or FLAG-VAPB KMDD mutant followed by serum starvation and IGF stimulation. Western blot was performed to measure the phosphorylation levels of pAKT (S473). Data in the bar graphs represent the mean ± SEM values of the ratios of densities for three independent experiments. **P < 0.01.

We next verified the physiological roles of the VAPB-IRS-1 axis in vivo. To further validate the roles of VAPB in regulating IRS-1 expression, we created a VAPB ablation mouse model (VAPB-KO) (Fig. 6a). Ablation of VAPB expression levels was verified by PCR on genomic DNA and Western blot of various tissues (Fig. 6b, c). Body weights of 6–8-week-old VAPB-KO mice did not differ from those of wild-type (WT) controls (Fig. 6d). Compared to the WT littermates, VAPB-KO mice exhibited decreased levels of IRS-1 in tissues, such as tibialis anterior (TA) and gastrocnemius (GAS) muscles (Fig. 6e, f). This occurred with no changes in IRS-1 expression at the mRNA level (Fig. 6g). We next measured the insulin signaling in vivo. The reduction of IRS-1 in protein levels was associated with a marked suppression of insulin-stimulated S473 phosphorylation of AKT in both TA and GAS (Fig. 6h, i). Glucose and insulin tolerance tests (GTT, ITT) were performed at 5-month-old mice fed with a regular chow diet. Comparing to WT mice, VAPB-KO mice were significantly glucose intolerant (Fig. 6j). ITT assay also revealed reduction in insulin sensitivity of chow-fed VAPB-KO mice (Fig. 6k). To verify the roles of VAPB-mediated ER-targeting of IRS-1 in regulating insulin signaling, we isolated hepatocytes form VAPB-KO mice and performed rescue assay using WT VAPB or the IRS-1 binding deficient KMDD mutant. Indeed, reintroducing of VAPB, but not the KMDD mutant, rescued the insulin-stimulated AKT S473 phosphorylation (Fig. 6l). All these in vivo results validated the essential roles of VAPB in regulating IRS-1 expression and insulin signaling.

### An amyotrophic lateral sclerosis (ALS)-derived VAPB mutant impairs IRS-1 stability and ER-IRS-1 tethering

**Fig. 7:**
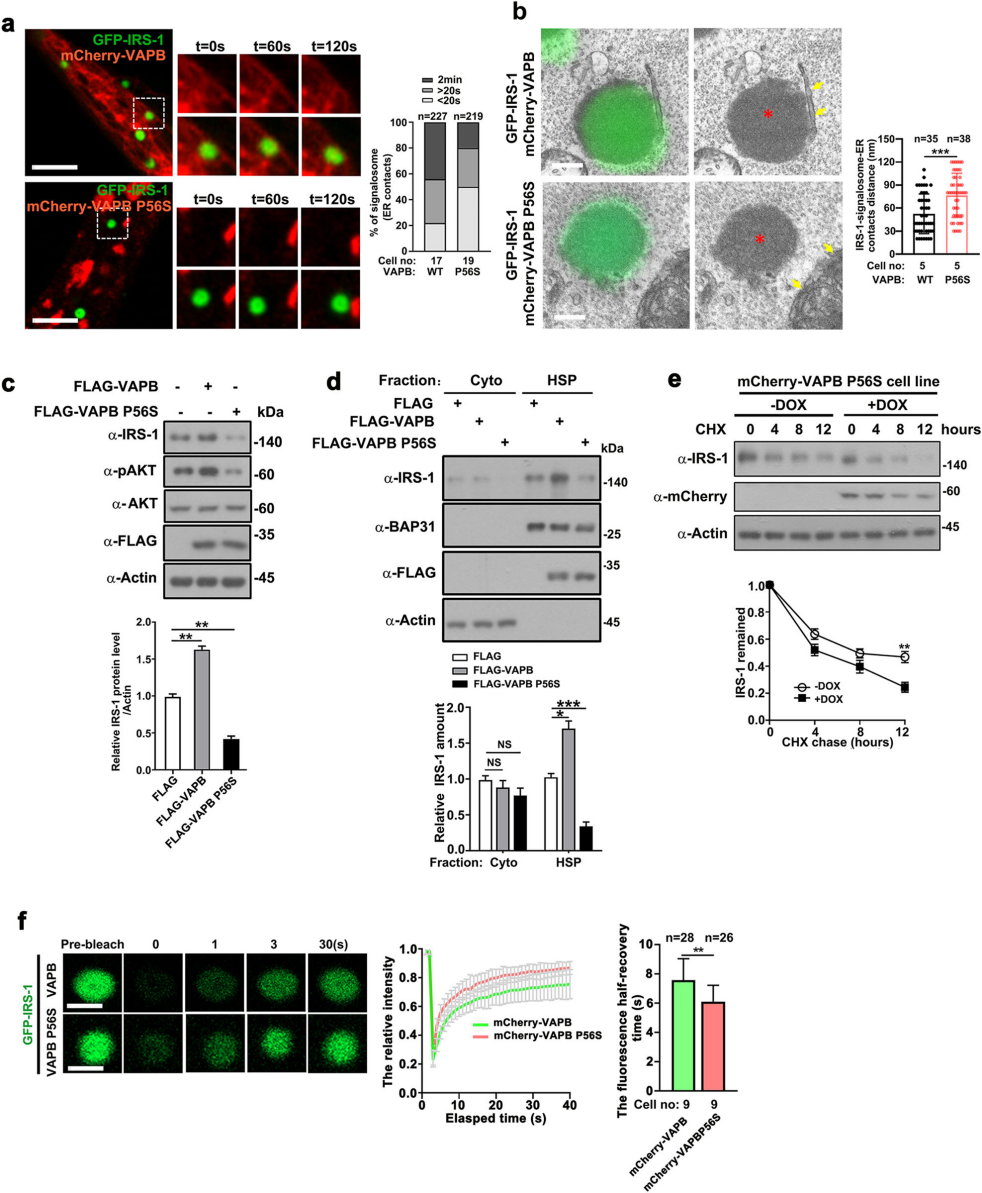
The ALS-derived VAPB P56S mutant impairs stability and ER- tethering of IRS-1. **a** Representative live imaging of GFP-IRS-1 droplets and mCherry-VAPB or mCherry-VAPB P56S mutant in C2C12 cells. Cells were quantified for the degree of association between IRS-1 signalosomes and VAPB WT or P56S mutant. Scale bars, 4 µm. **b** CLEM of C2C12 cells expressing GFP-IRS-1 and mCherry-VAPB or mCherry-VAPB P56S. Scale bars, 0.4 µm. Red asterisks (*) indicate nonmembrane GFP-IRS-1 foci while yellow arrows indicate ER. Minimum distance of ER located from the considered IRS-1 foci was quantified. Data are represented as mean ± SD. ***P < 0.001. **c** Lysates of C2C12 myoblasts transfected with control FLAG vector, FLAG-VAPB, or FLAG-VAPB P56S mutant were western blotted for assessing expression levels of IRS-1 and phosphorylation levels of AKT. Data in the bar graphs represent the mean ± SEM values of the ratios of densities for three independent experiments. **P < 0.01. **d** C2C12 myoblasts stably expressing control FLAG vector, FLAG-VAPB, or FLAG-VAPB P56S mutant were homogenized and extracts were fractionated into cytosol and HSP. Fractions were analyzed by western blotting and quantified. Data in the bar graphs represent the mean ± SEM values of the ratios of densities for three independent experiments. *P < 0.05, ***P < 0.001. ns: not significant. **e** CHX chase analysis. Cells were treated with CHX (25 µg/mL) and chased for times as indicated. Data are represented as mean ± SEM. **P < 0.01. **f** Confocal imaging of IRS-1 fluorescence recovery after photobleaching in C2C12 cells co-expressing GFP-IRS-1 and mCherry-VAPB or mCherry-VAPB P56S. Scale bars, 1 µm. Right panel: quantification of fluorescence intensity recovery of photobleached IRS-1 bodies (*n* = 12). Data are represented as mean ± SD. The recovery half time of GFP-IRS-1 foci were measured. Data are represented as mean ± SD. **P < 0.01.

The dominant proline-to-serine missense mutation in VAPB (P56S) is linked to the familial motor neuron disease, ALS^47^. Overexpression of this mutant causes ER clustering and functional disorders^47^. To validate the essential roles of VAPB in coordinating the ER membrane association of IRS-1, we tested the effect of the ALS-derived VAPB P56S mutant on IRS-1 location and turnover. It is previously reported that P56S mutant perturbs FFAT-motif binding and traps endogenous VAP in mutant aggregates^48,49^. In line with these previous findings, we found that the P56S mutant reduced the interaction between endogenous IRS-1 and VAPB (Supplementary Fig. S9a). Comparing to WT VAPB, the P56S mutant displayed an impaired IRS-1-binding ability (Supplementary Fig. S9b). Consistently, the P56S mutation abolished the tethering between VAPB and IRS-1 (Fig. 7a; Supplementary Fig. S9c). CLEM also revealed impaired ER-contact of IRS-1 condensates in VAPB P56S expressing cells (Fig. 7b). Notably, the expression of IRS-1, as well as AKT phosphorylation, was significantly suppressed in P56S expressing cells (Fig. 7c). Correspondingly, upon the expression of VAPB P56S, the intracellular membrane-targeted IRS-1 was evidently reduced (Fig. 7d). Correspondingly, the P56S impaired the stability of endogenous IRS-1 (Fig. 7e). Cytosolic condensates displayed a more liquid-like behavior^8^. Indeed, the expression of P56S elevated the FRAP rate of IRS-1 (Fig. 7f), indicating a detachment of IRS-1 droplets from ER membrane. These results indicate that the ALS-associated P56S mutant enhanced IRS-1 turnover, possibly by interfering with the CMC between ER and IRS-1.

## Discussion

**Fig. 8:**
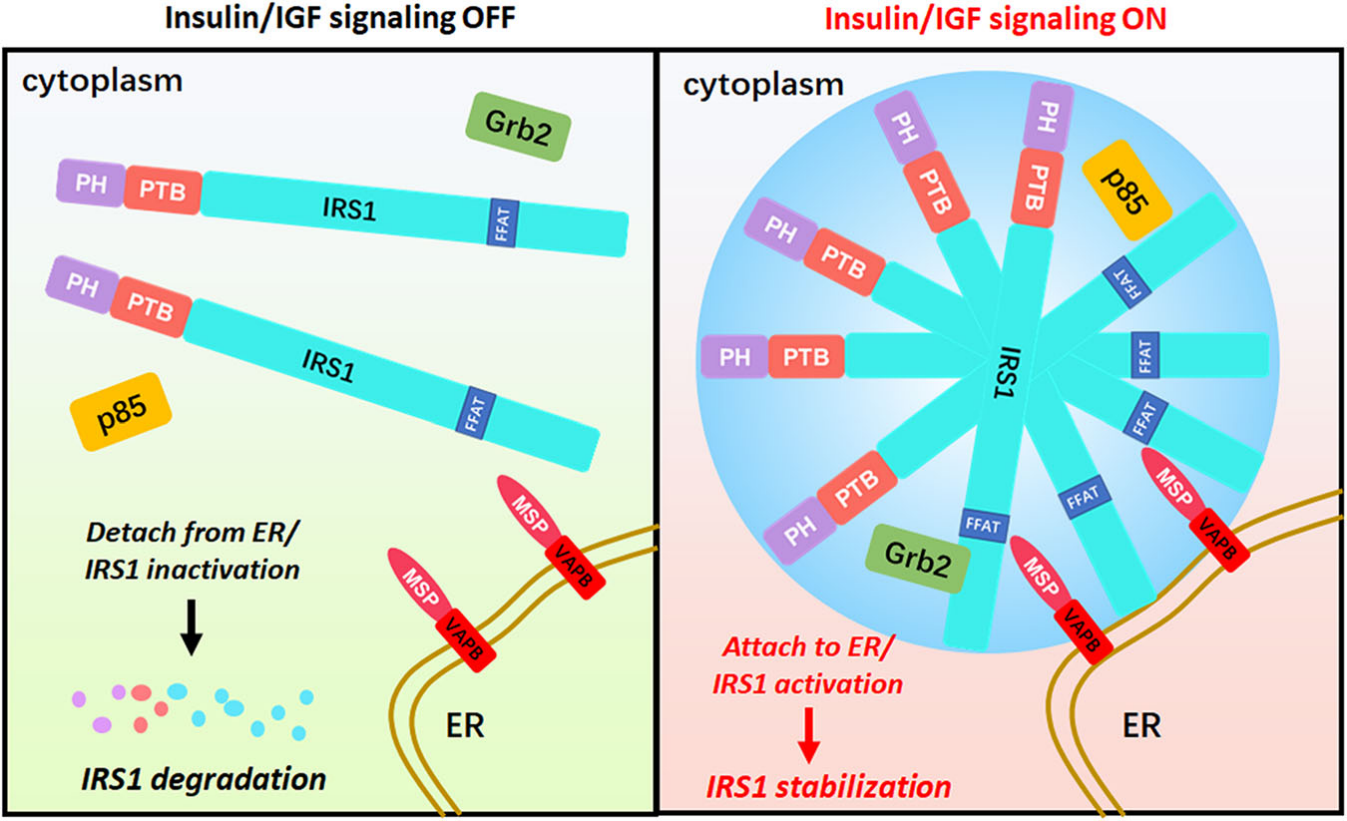
Schematic model for VAPB-mediated ER-tethering of IRS-1 signalosomes. We found that IRS-1 signalosomes attach to the ER membrane through interacting with VAPB. The FFAT motif in IRS-1 binds the MSP domain of VAPB. Insulin/IGF signaling promotes the IRS-1–VAPB interaction and subsequently stabilizes IRS-1.

In addition to forming nuclear and cytoplasmic compartments, biomolecule condensation/phase separation enables essential scaffold proteins to assemble signalosomes, which create a high local concentration of signaling components and promote the signaling outputs^50–58^. However, the mechanisms by which cells modulate functional signalosomes at the appropriate cellular membrane location remains largely unexplored. We and another group have recently revealed that the spherical IRS-1 foci have a highly dynamic liquid-like nature, and recruit downstream effectors, including PI3K and Grb2, to form insulin/IGF signalosomes^29,59^. In the current paper, we demonstrated that IRS-1-mediated signalosomes interact with ER in a VAPB-dependent manner (Fig. 8). Therefore, our findings reveal that, beside generating tethers between ER and other organelle membranes^4,10^, VAPB also mediates CMC. Notably, ablation of VAPB impaired insulin signaling by downregulating the IRS-1 expression levels and AKT activation in vivo (Fig. 6). Since ER governs protein synthesis and folding, this ER-IRS-1 association represents a mechanism to ensure that insulin/IGF is active only in cells maintaining protein homeostasis.

The connection between endomembrane and IRS-1 stability and activation is well recognized^31–34,46^. Our findings suggest that the VAPB-mediated ER-association with IRS-1 appears to be the major, if not the only, mechanism of IRS-1 activation and stability (Fig. 8). This is in line with the previous finding that ER stress leads to IRS inactivation^60^. We may ask, why should the ER regulate the IRS-1 activation? As a vast membranous network, the ER is responsible for the synthesis, maturation, and trafficking of proteins^4^, while insulin/IGF signaling regulates metabolism, differentiation, and the growth of cells^61–63^. Therefore, the modulation of IRS-1 by the ER ensures that the insulin/IGF signaling is not inappropriately activated in cells with aberrant protein synthesis. In support of this notion, we observed that ER-stress led to detachment of IRS-1 puncta from ER (Fig. 1f, g, h). Indeed, ER stress suppressed the mTOR complex 1 (mTORC1)^64,65^, which governs protein translation. Our findings indicate that the effect of ER stress on mTORC1 is due, at least partially, to the inhibition of IRS-1-mediated insulin/IGF signalosomes. Overall, the ER appears to be a signaling platform to coordinate insulin/IGF and protein synthesis.

The membrane surface has long been recognized to increase the local concentration of signaling molecules by restricting protein diffusion into two dimensions^66^. Moreover, it is recently becoming appreciated that the membrane surface also acts as a pivotal regulatory platform for controlling phase separation/biomolecular condensation^5,8,67^. Two of the best characterized examples are linkers for the activation of T cell (LAT)-based phase separation and phase separated network of nephrin-Nck-N-WASP^68–70^. In addition to the plasma membrane, endomembrane surfaces have recently been recognized as an organizing platform for biomolecule condensates^39,71–73^. For example, ER and RNA-binding protein TIS11B form a meshwork to facilitate the protein–protein interactions required for protein trafficking^71^. Interestingly, ER membranes exhibit phase behavior at sites of organelle contact^72^. Notably, ER membrane-tethered Whi3 displayed a low mobile protein fraction^73^. This is in line with our findings showing that the IRS-1 puncta detached from ER are more liquid-like as shown in the FRAP assay (Supplementary Fig. S3). Since insulin and IGF-1 normally act through their receptors, which locate at plasma membrane, it is thus of interest to illustrate how insulin and IGF-1 can activate the IRS-1 signalosome located at ER. Rab5 could contribute to this linkage because it regulates endocytosis, the cellular events that controls internalization of plasma membrane receptors^74^. In line with this hypothesis, we have reported that Rab5 determines the interaction between IGFR and IRS-1^36^. Interestingly, we have also demonstrated that Rab5-positive endosome displays attachment with IRS-1 puncta^29^ (Supplementary Fig. S1b). More work is needed to understand the cross-talk between endosome and the ER-IRS-1 tethering.

Aberrant phase separation results in ALS diseases^75–77^. This includes the disease mutations of FUS, hnRNPA2, and TDP-43, which drive the liquid condensates into more solid-like states^78–80^. Single amino acid mutation in VAPB causes ALS-type 8^47^. In addition to the progressive degeneration of motor neurons, ALS has also been shown to be associated with defective energy metabolism such as weight loss, hyperlipidaemia, and glucose intolerance^81,82^. Therefore, ALS is thought to be a systemic disease and alteration of energy homeostasis in ALS might contribute targets for novel therapeutic strategies. Likewise, the ablation of VAPB in mice, or the VAPB homolog in *C. elegans* led to metabolic defection^83^. Here, we demonstrate that ALS-derived VAPB mutation disrupts the tethering with IRS-1 droplets (Fig. 7a, b), reduces the intracellular membrane-containing fraction of IRS-1 (Fig. 7d), and leads to the degradation of IRS-1 (Fig. 7e). It is noteworthy that ER stress, which was observed in P56S knock-in mice^84^, impaired the endomembrane-tethering of IRS-1 (Fig. 1f, g, h). We thus propose that the pathological effects of the P56S mutation might be, at least partially, due to the impairment of VAPB mediated ER-tethering of IRS-1. These results thus strongly implicate the spatial regulation of IRS-1 in ALS-associated metabolic defects and this observation may provide new opportunities for therapeutic interventions.

## Materials and methods

### Culture and maintenance of cells

C2C12 cell line was from Cell bank of the Chinese Academy of Sciences; HEK293T cell line was from American Type Culture Collection. C2C12 cells were grown in DMEM (high glucose) supplemented with 15% (v/v) fetal bovine serum and human 293T cells were grown in RPMI-1640 medium supplemented with 10% (v/v) fetal bovine serum, 2 mM L-glutamine, 100 U/mL penicillin and 100 mg/mL streptomycin (all from Hyclone Laboratories, Logan, UT).

### IGF-1 stimulation and ER stress induction

C2C12 cells were serum-starved for 12 h in DMEM, and then treated with 100 ng/mL IGF-1 (Sino biological, 10598-HNAY1). Thapsigargin (ALADDIN, T135258) and Tunicamycin (ALADDIN, T101151) were used to induce ER stress for indicated time.

### Antibodies

The following antibodies were purchased from Cell Signaling Technology: anti-IRS-1 (2382), anti-pAKT S473 (9271), anti-p85 (4292), anti-IGF-1Rβ (9750), anti-PDK1 (13037). Other antibodies were from the following commercial sources: anti-VAPB (Sigma-Aldrich, HPA013144), anti-NOGOA (Bio-Rad, AHP1799), anti-BAP31 (Santa Cruz Biotechnology, sc-48766), anti-PERK (abcam, ab229912), anti-pPERK (Affinity Biosciences, DF7576), anti-AKT (HUABIO, ET1609-47), anti-Actin (HUABIO, M1210-2), anti-Tubulin (HUABIO, M1305-2), anti-GFP (HUABIO, ET1607-31), anti-FLAG (YEASEN, 30503ES60), anti-HA (Invitrogen, PA1-985) and anti-mCherry (ABclonal, AE002).

### Plasmids

Full-length cDNAs of IRS-1, VAPB and mutant proteins were cloned into a hemagglutinin (HA)-tagged, GFP-tagged, and FLAG-tagged pXJ40 expression vector (E Manser, IMCB, Singapore). All plasmids were purified using an Axygen miniprep kit for use in transfection experiments. *Escherichia coli* strain DH5-α was used as a host for propagation of the clone. All the mutations used in this study were created using the standard PCR-based mutagenesis method and confirmed by DNA sequencing.

### Mice and animal care

Mouse strains obtained from Cyagen (Suzhou, China) are under the stock number: KOCMP-56491-Vapb-B6N-VA (VAPB-KO, C57BL/6N-Vapb^em1cyagen^). PCR genotyping was done by using protocols described by the supplier. Mice with same age and without stress or discomfort signs (including stereotyped behaviors and hair loss) were employed to minimize physiological variability. All mice used in this study had a C57BL/6N genetic background and were housed in a pathogen-free facility in the University Laboratory Animal Center. All animal experiment protocols were approved by the Review Committee of Zhejiang University School of Medicine and were in compliance with ethical regulations and institutional guidelines.

### Glucose and insulin tolerance test

For glucose tolerance test (GTT), mice were fasted for 12 h, and then injected intraperitoneally with glucose (Sigma-Aldrich, G5767) saline solution (1.5 g/kg body weight). For insulin tolerance test (ITT), mice were fasted for 6 h and injected intraperitoneally with insulin (Novolin R, HH20170016) saline solution (1 U/kg of body weight). Blood glucose levels were measured by tail-snip blood sampling pre-injection and 15-, 30-, 60-, and 120-min after injection.

### Mouse primary hepatocyte isolation/culture

Primary hepatocytes were isolated from 6–8-week-old male mice as previously described^85^. Briefly, after the mice were anesthetized, they were perfused through the portal vein with perfusion medium (Life Technologies, 17701-038) followed by liver digest medium (Life Technologies, 17703-034). Next, the liver was excised, minced and filtered through a 100 mm steel mesh, and hepatocytes were separated by two times of centrifugations at 50× *g* for 1 min. The obtained hepatocytes were cultured in DMEM medium containing 10% fetal bovine serum and 1% penicillin-streptomycin in a 5% CO_2_/water-saturated incubator. Transfection of primary hepatocytes was performed with Hieff Trans^TM^ Universal Transfection Reagent (Yeasen Biotechnology, #40808ES).

### Live-cell imaging

C2C12 cells were seeded onto 8-well chamber slides (Cellvis, C8-1.5H-N) and plasmid transfections were performed with lipofectamine 3000 (Invitrogen, L3000015). After 24 h incubation, live-cell imaging was performed at 37 °C and images were captured with the Zeiss LSM 880 equipped with Airyscan detectors and 63×/1.4-NA plan Apochromat oil objective using Zeiss ZEN software. For tethering experiment, 2-min time-lapse movies of C2C12 cells transfected with GFP-IRS-1 and mCherry-VAPB or mCherry-Sec61β (fluorescent markers for the ER) were collected as previously described^39^. The IRS-1 signalosome was considered as contacting with ER when the value of Mander’s overlap coefficient was > 0.1. IRS-1 signalosome contact with the ER was classified into three categories: The droplet contacts the ER for (i) < 20 s or not at all, (ii) at least 20 s but < 2 min, and (iii) the entire 2-min movie. Image acquisition and analysis for tethering of IRS-1 condensates and VAPB or ER were blinded.

### FRAP

FRAP experiments were performed on a Zeiss 800 microscope with a 63× oil immersion objective. C2C12 cells were seeded onto 8-well chamber slides (Cellvis, C8-1.5H-N). DNA plasmid transfections were performed with lipofectamine 3000 (Invitrogen, L3000015). After 24 h incubation, GFP-IRS-1 droplets were photobleached using a laser intensity of 80% at 480 nm (for GFP) and recovery was recorded for the indicated time. The prebleached fluorescence intensity was normalized to 1 and the signal after bleach was normalized to the prebleach level.

### Direct binding assay

293 T lysates transfected with FLAG-tagged expression plasmids were subjected to immunoprecipitation with anti-FLAG M2 affinity gel (Bimake, B23102). The desired proteins were eluted from the beads with 200 ng/μL FLAG peptide (MCE, HY-P0319). Subsequently GST control or GST fusion protein-beads were incubated with eluted FLAG-tagged proteins at 4 °C for 2–3 h in binding buffer (200 mM sodium chloride, 50 mM Tris, pH 7.3, 0.25 mM EDTA, 1% (w/v) sodium deoxycholate, 1% (v/v) Triton X-100, 0.2% sodium fluoride, 0.1% sodium orthovanadate, and a mixture of protease inhibitors (Selleck Chemicals, B14001)). The bound proteins were separated by SDS-PAGE for Western blot using anti-FLAG antibody.

### Generation of inducible stable cell line

A tet-on system was used for C2C12 cells to generate inducible stable cell lines as previously described. Cells were co-transfected with HP216 vector and HP138-GFP-IRS-1 or HP138-mCherry-VAPB P56S vector. After 24 h, cells were treated with 500 ng/mL doxycycline (YEASEN, 60204ES03) for 1 day to induce the expression of proteins and sorted by flow cytometric fluorescence sorting (Beckman moflo Astrios EQ). Western blot was performed to validate the expression levels.

### Quantitative real-time PCR

As previously described^86^, total RNA was isolated using RNeasy Kit (Axygen, AP-MX-MS-RNA-250) and reverse transcription was performed using a SuperScript III reverse transcriptase kit (Vazyme, R223-01). qPCR reactions using SYBR qPCR Master Mix kit (Vazyme, R711-02) were performed in triplicate for each gene with three independent samples prepared under the same conditions. Values were calculated using the second derivative method and normalized to *Actin* expression. The relative levels of mRNAs were calculated according to the 2^-(ΔΔCt)^ equation. The primers for qPCR are listed below:

*IRS-1* forward-1: 5'- ACGAACACTTTGCCATTGCC-3';

*IRS-1* reverse-1: 5'- CCTTTGCCCGATTATGCAGC-3';

*IRS-1* forward-2: 5'- CTCCTGCTAACATCCACCTTG-3';

*IRS-1* reverse-2: 5'- AGCTCGCTAACTGAGATAGTCAT-3';

*Actin* forward: 5'-ATGCTCCCCGGGCTGTAT -3';

*Actin* reverse: 5'-CATAGGAGTCCTTCTGACCCATTC-3'.

### RNA interference

C2C12 myoblasts were transfected with siRNA oligoes using Lipofectamine RNAiMAX (Invitrogen, 13778150) at 30%–40% confluency according to the manufacturer’s protocol. The siRNA oligos used in this study are listed below:

Control siRNA: 5'- UUCUCCGAACGUGUCACGU-3'

*VAPB* siRNA1: 5'-CAGUAUGGAAGGAGGCAAA-3'

*VAPB* siRNA2: 5'- GCAACCCAACAGACCGAAA-3'

### Immunoprecipitation studies and Western blot analyses

Control cells or cells transfected with expression plasmids were lysed in lysis buffer (150 mM sodium chloride, 50 mM Tris, pH 7.3, 0.25 mM EDTA, 1% (w/v) sodium deoxycholate, 1% (v/v) Triton X-100, 0.2% sodium fluoride, 0.1% sodium orthovanadate, and a mixture of protease inhibitors from Roche Applied Science). Lysates were immunoprecipitated (IP) with anti-FLAG M2 beads (Bimake, B23102) or protein A/G agarose resin (YEASEN, 36403ES25). Samples were run in SDS/PAGE gels and analyzed by Western blotting with anti-FLAG or indicated antibodies.

### Immunofluorescence and direct fluorescence studies

Cells were seeded on coverslips in a six-well plate and transfected with various expression constructs for 24–36 h and then stained for immunofluorescence detection using confocal fluorescence microscopy or directly visualized for cells expressing GFP-tagged proteins as previously described^87^. The images were collected with a 63 × 1.4 NA or 20× objective lens using appropriate laser excitation on a LSM800 Meta laser-scanning confocal microscope (Carl Zeiss). The detector gain was first optimized by sampling various regions of the coverslip and then fixed for each specified channel. Once set, the detector gain value was kept constant throughout the image acquisition process. Images were analyzed with Zeiss LSM Image Examiner Software. As previously described^39^, colocalization between IRS-1 puncta and ER tubules was determined by calculating the Mander’s coefficient of the percentage of IRS-1 condensates overlapping with ER tubules.

### Correlative confocal and electron microscopy

C2C12 cells were plated on glass gridded coverslips (Cellvis, D35-14-1.5GI) and transfected with indicated plasmids. The cell density is controlled at 50% to clearly observe the location coordinates of the cells under light microscopy. The cells were fixed with 3% paraformaldehyde for 20 min and imaged on Zeiss Airyscan to collect light microscopy images. During the process of images collection, the location coordinates of each target cell were recorded and the brightfield and fluorescence images of the target cells under low magnification were captured, both of which facilitate rapid searching under electron microscopy in the following steps. The cells were then fixed with 2.5% glutaraldehyde for 12 h at 4 °C and postfixed in 2% osmium tetroxide-3% potassium ferrocyanide in cacodylate buffer for 1 h followed by 1% thiocarbohydrazide dissolved in water for 20 min and incubated in 2% osmium in cacodylate buffer for 30 min. Samples were then dehydrated with a graded ethanol series (20%, 50%, 70%, 90%, and 100%) for 15 min each and processed for Epon embedding. The samples were cut (30 KV and 2.5 nA) and imaged (2 KV and 0.2 nA) by FIB-SEM (Helios UC G3).

### Subcellular fractionation

The subcellular fractionation experiments were carried out as previously described^46^. Briefly, cells were washed with ice-cold PBS and homogenized in buffer (0.5 mM EDTA, 10 mM Tris (pH 7.4), 25 mM sucrose, supplemented with protease and phosphatase inhibitors) by Dounce homogenizer with 100 strokes. The homogenate was centrifuged at 800× *g* for 10 min at 4 °C to obtain LSP fractions. The supernatant was then recentrifuged at 200,000× *g* for 1 h at 4 °C to acquire cytosol and HSP fractions. The HSP and LSP fractions were resuspended with an IP buffer (20 mM Tris (pH 7.5), 1 mM EDTA, 150 mM NaCl, 1% Triton X-100, 1 mM EGTA) and RIPA, respectively.

### Three-dimensional (3D) rendering

Z stack images were acquired using a Zeiss LSM 800 confocal microscope. The step size was 0.2 µm. 3D rendering was performed using Imaris software. Z-stack images of cells were captured within under identical conditions with respect to laser intensities and exposures.

### Statistical analysis

Statistical analyses were performed in GraphPad Prism 8.0.2 (GraphPad Software, Inc.). Results are presented as mean ± SEM. or mean ± SD. Statistical significance was determined as indicated in the figure legends: *P < 0.05, **P < 0.01, ***P < 0.001, ****P < 0.0001. The data distribution was first checked using a Shapiro-Wilk normality test, Kolmogorov–Smirnov test and D’Agostino & Pearson omnibus normality test. For comparison between two groups and if the data fitted a normal distribution, a two-tailed unpaired Student’s *t*-test was used when variances were confirmed as similar via an *F*-test (*P* > 0.05). A two-tailed unpaired Student’s *t*-test with Welch’s correction was used when variances were shown up as different via the *F*-test (*P* < 0.05). If the data did not fit a normal distribution, a Mann–Whitney test was used. If the variation among three or more groups was minimal, ANOVA followed by Dunnett’s post-test or Tukey’s post hoc test was applied for comparison of multiple groups.

## Supplementary information

Supplemental Figures
