# Supplementary material for: VAPB-mediated ER-targeting stabilizes IRS-1 signalosomes to regulate insulin/IGF signaling
Source: Cell Discov. 2023 Aug 1;9:83. doi: 10.1038/s41421-023-00576-6 (PMC10394085; doi:10.1038/s41421-023-00576-6)
Supplement: Supplementary file 1 — Supplemental Figures [file 41421_2023_576_MOESM1_ESM.pdf]

Supplemental Figures and Legends

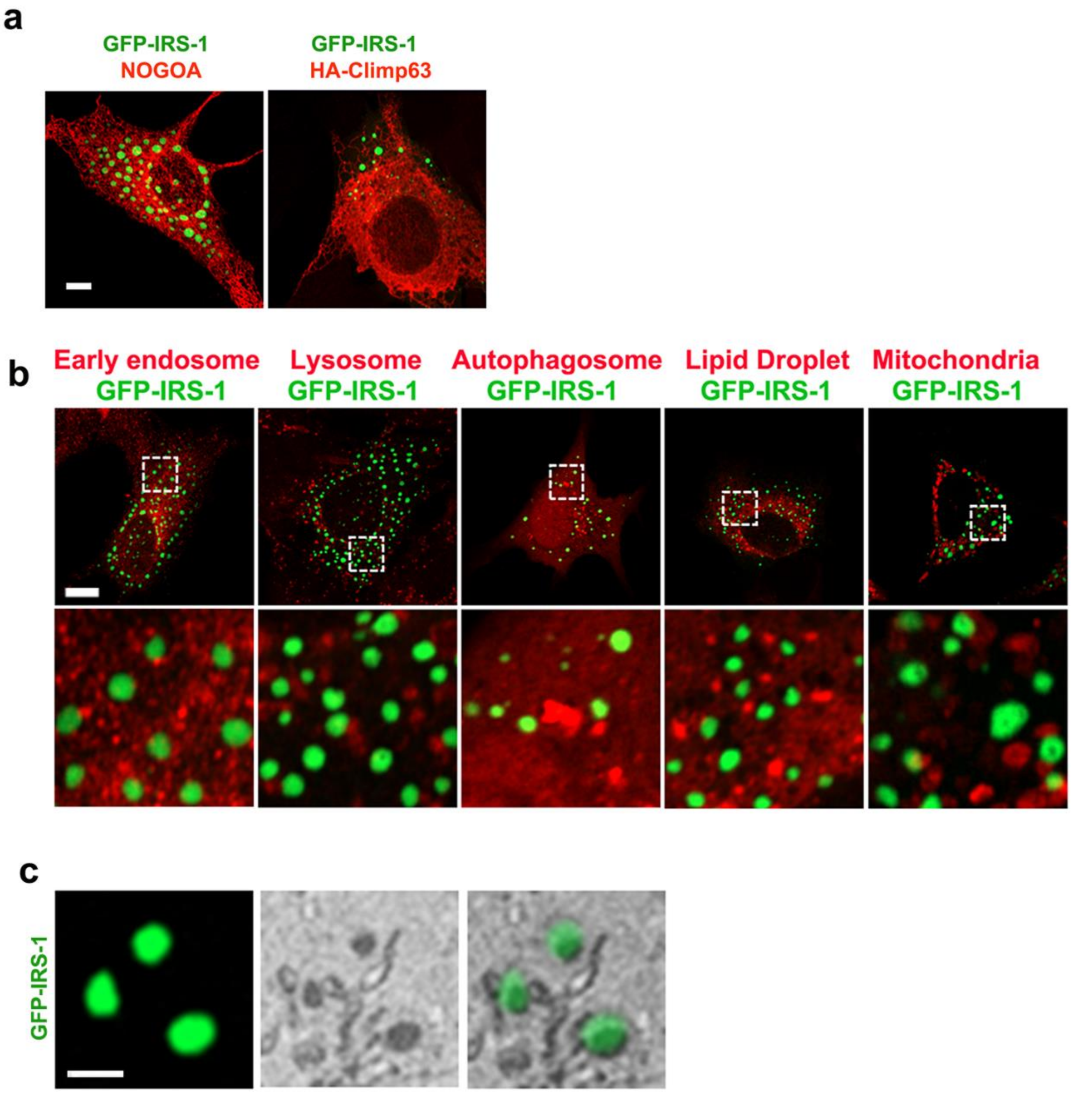

Fig S1. The membraneless IRS-1 condensates are associated with ER.

a Z-stacking confocal images of representative GFP-IRS-1 C2C12 myoblasts stained with endogenous NOGOA (left panel) or HA-Climp63 (right panel). Scale bar, 5 µm. **b** Representative confocal images of GFP-IRS-1 and endogenous Rab5 (for early endosome), Lamp1 (for lysosome), RFP-LC3 (autophagosome), mCherry-LiveDrop (lipid droplet), or FLAG-ANT2 (mitochondria). Scale bar, 5 µm. **c** Correlative light and electron microscopy (CLEM) of C2C12 cells expressing GFP-IRS-1. Scale bar, 1 µm.

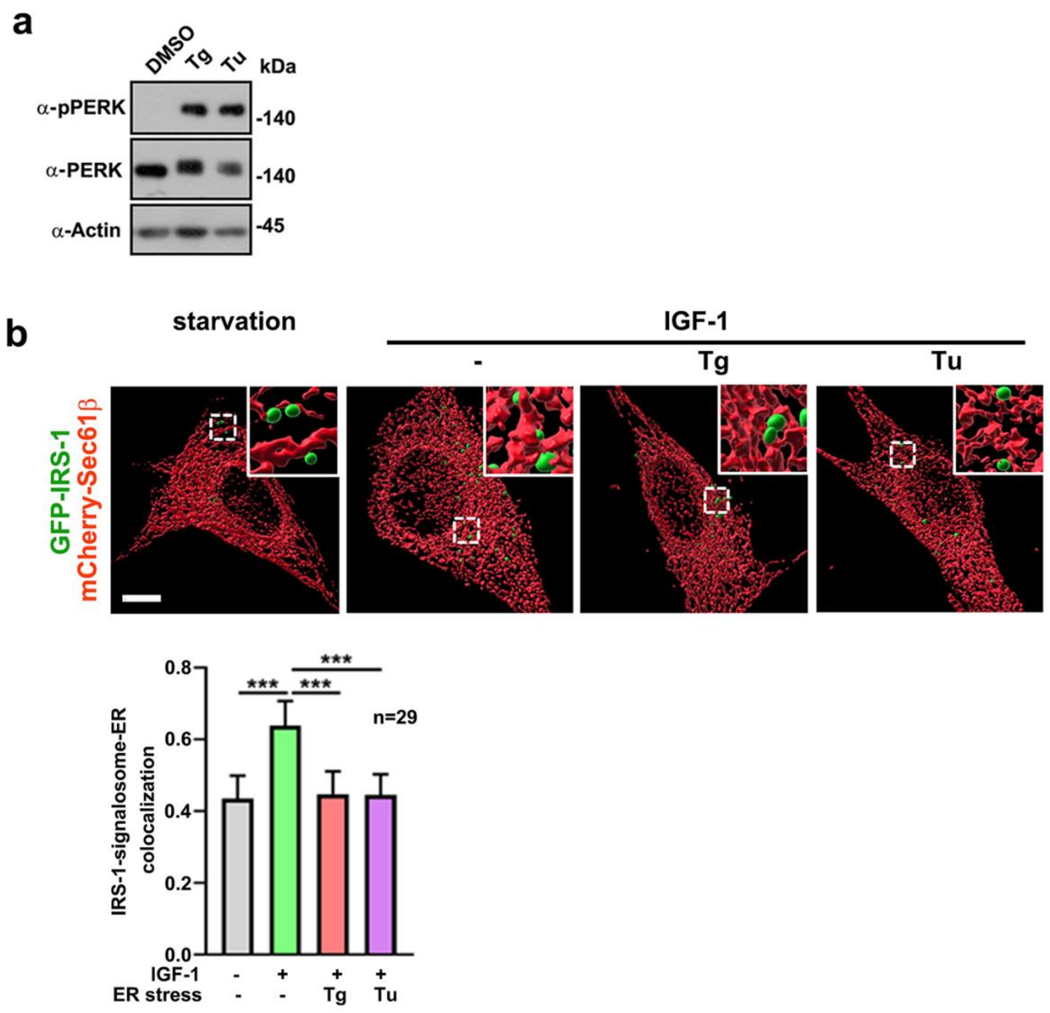

Fig S2. ER stress impairs ER tethering of IRS-1 foci.

a Lysates of C2C12 myoblasts treated with DMSO, tunicamycin (Tu), or thapsigargin (Tg) were western blotted with the indicated antibodies for assessing ER stress. **b** C2C12 expressing GFP-IRS-1 were serum starved for 16 hours followed by treating with DMSO, Tu (2μg/mL), or Tg (1μM) for 3 hours. Cells were collected for immunostaining after IGF-1 stimulation (2.5 min) and the ER-attachment of IRS-1 signalosome were quantified. ***: p<0.001. Scale bar, 5 μm.

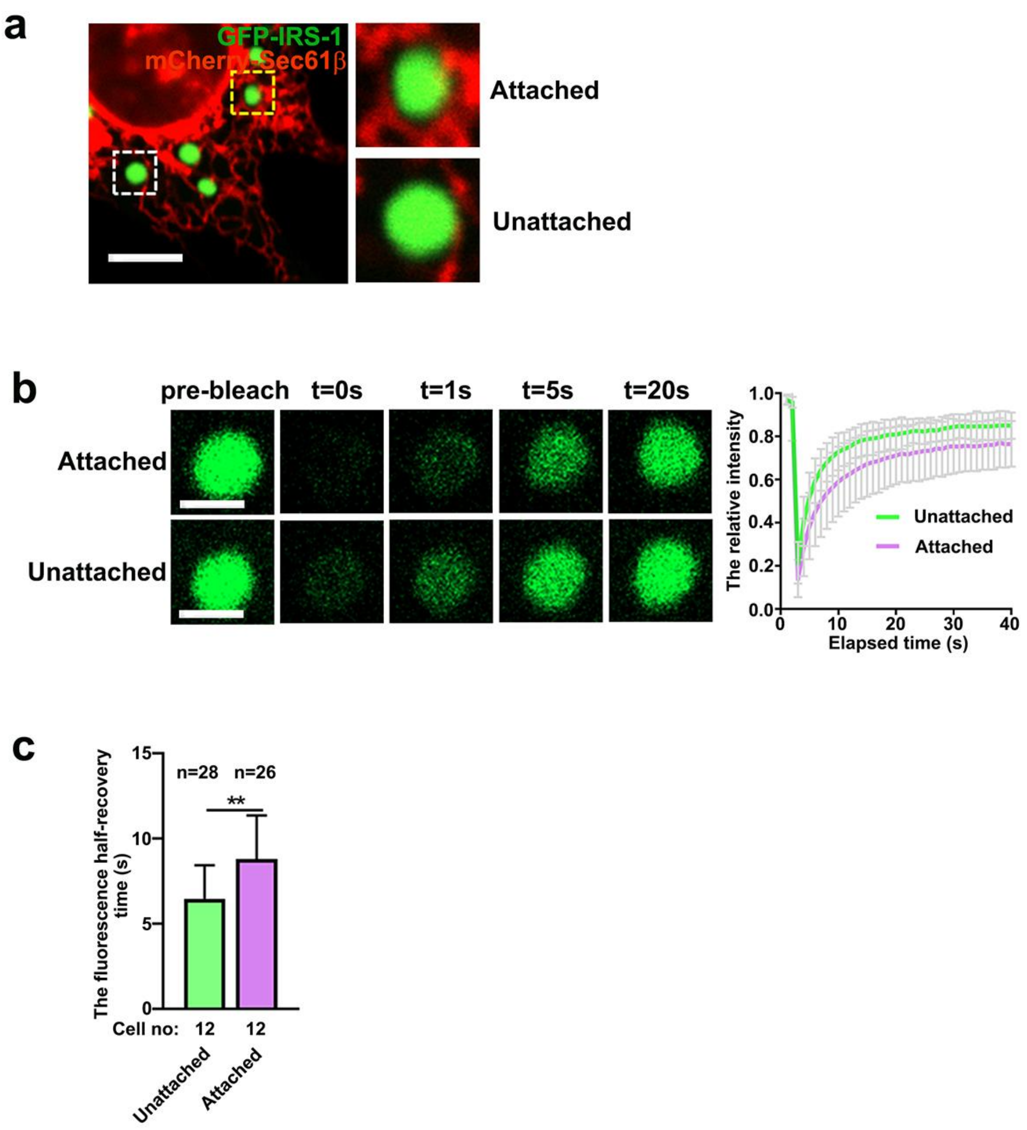

Fig. S3. The IRS-1 condensates attached to ER membrane display a more liquid-like behavior.

a Representative live imaging of GFP-IRS-1 droplets attached or unattached to ER membrane (mCherry-Sec61β) in C2C12 cells. Scale bar, 4 μm. **b** FRAP analysis of GFP-IRS-1 droplets attached or unattached to ER membrane. Scale bar, 1 μm. **c** The recovery half time of GFP-IRS-1 foci attached or unattached to ER membrane were measured. **: $p < 0.01$.

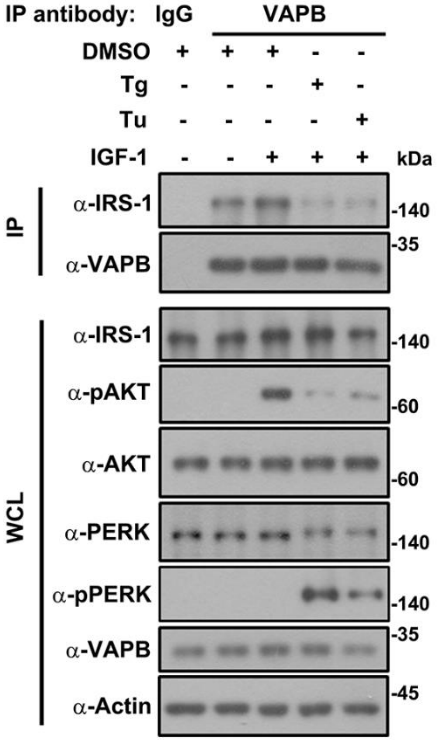

Fig. S4. Tg or Tu treatment impaired the VAPB-IRS-1 association.

C2C12 myoblasts were serum starved for 16 hours followed by treating with DMSO, Tu (2μg/mL), or Tg (1μM) for 3 hours. Cells were then stimulated with IGF-1 for 2.5 min and subjected to immunoprecipitation with VAPB antibodies. Coimmunoprecipitated IRS-1 and VAPB were detected by Western blot analysis.

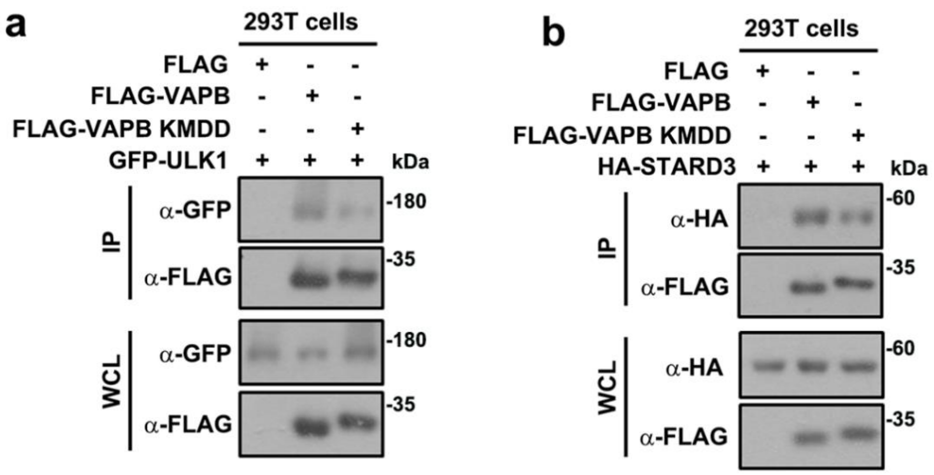

Fig. S5. VAPB interacts with FFAT motif-containing proteins.

a FLAG-tagged VAPB or KMDD mutant was co-transfected with GFP-ULK1 into 293T cells for co-immunoprecipitation analysis. **b** FLAG-tagged VAPB or KMDD mutant was co-transfected with HA-STARD3 into 293T cells for co-immunoprecipitation analysis.

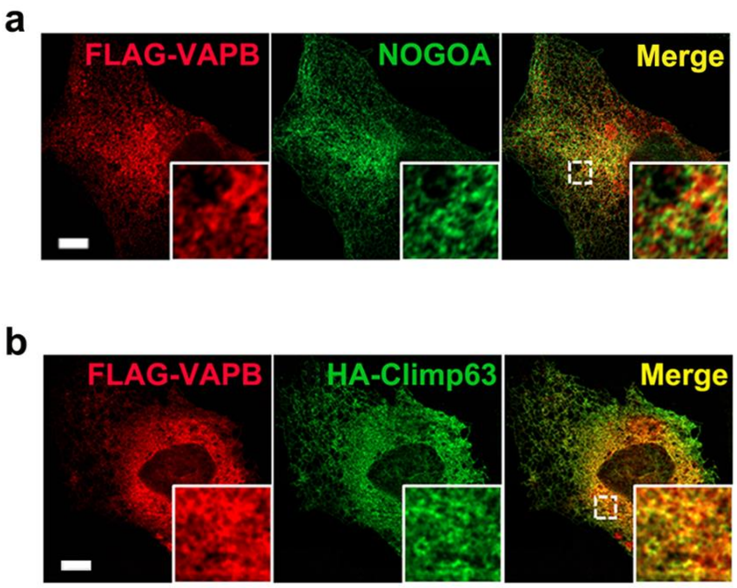

Fig S6. VAPB displayed typical ER-structure. a Immunofluorescence staining of endogenous NOGOA and exogenous FLAG-VAPB in C2C12 myoblasts. Scale bar, 5 μm. **b** Confocal images of representative C2C12 myoblasts co-expressing FLAG-VAPB and HA-Climp63. Scale bar, 5 μm.

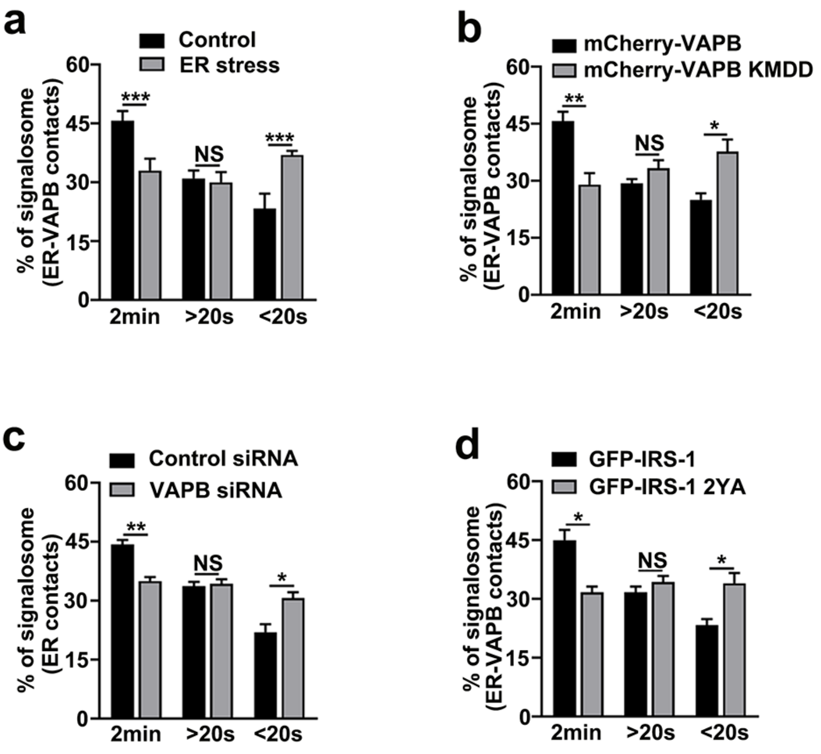

Fig S7. Quantification of association between IRS-1 signalosomes and ER or VAPB. **a** Quantification of association between VAPB and IRS-1 signalosomes in control and ER stress cells from 3 biologically independent samples (12 cells for each sample). n=110, 117, or 120 puncta from control cells and n=112, 121, or 125 puncta from ER stress cells. **b** Quantification of association between GFP-IRS-1 droplets and mCherry-VAPB or mCherry-VAPB KMDD mutant from 3 biologically independent samples (12 cells for each sample). n=115, 120, or 128 puncta from mCherry-VAPB transfected cells and n=108, 119, or 125 puncta from mCherry-VAPB KMDD mutant transfected cells. **c** Quantification of association between GFP-IRS-1 droplets and mCherry-Sec61β in control and VAPB-depleted cells from 3 biologically independent samples (12 cells for each sample). n=116, 121, or 126 puncta from control cells and n=109, 114, or 120 puncta from VAPB-depleted cells. **d** Quantification of association between

GFP-IRS-1 or GFP-IRS-1 2YA mutant droplets and mCherry-VAPB from 3 biologically independent samples (10 cells for each sample). n=98, 103, or 106 puncta from GFP-IRS-1 transfected cells and n=95, 100, or 105 puncta from GFP-IRS-1 2YA mutant transfected cells. *: $p < 0.05$, **: $p < 0.01$, ***: $p < 0.001$. NS: not significant.

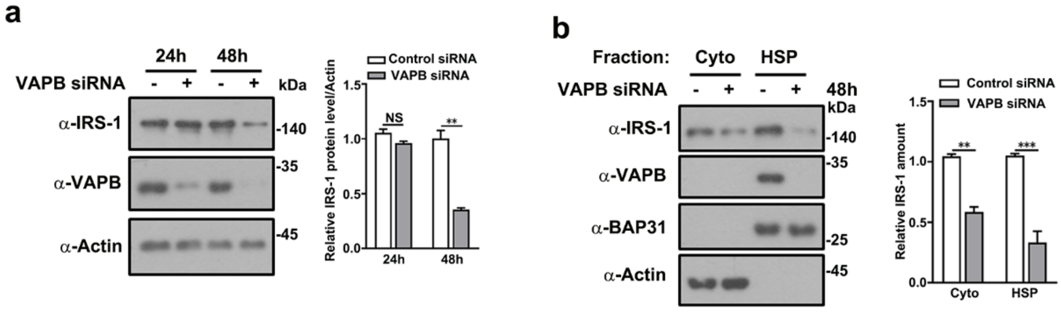

Fig. S8. Prolonged VAPB depletion time leads to IRS-1 degradation.

a. Lysates of C2C12 myoblasts transfected with either control or VAPB siRNA for 24 and 48 hours were western blotted with the indicated antibodies. **b.** C2C12 myoblasts transfected with control or VAPB siRNA for 48 hours were homogenized and extracts were fractionated into cytosol and HSP. Fractions were analyzed by western blotting with the indicated antibodies. **: $p < 0.01$, ***: $p < 0.001$. NS: not significant.

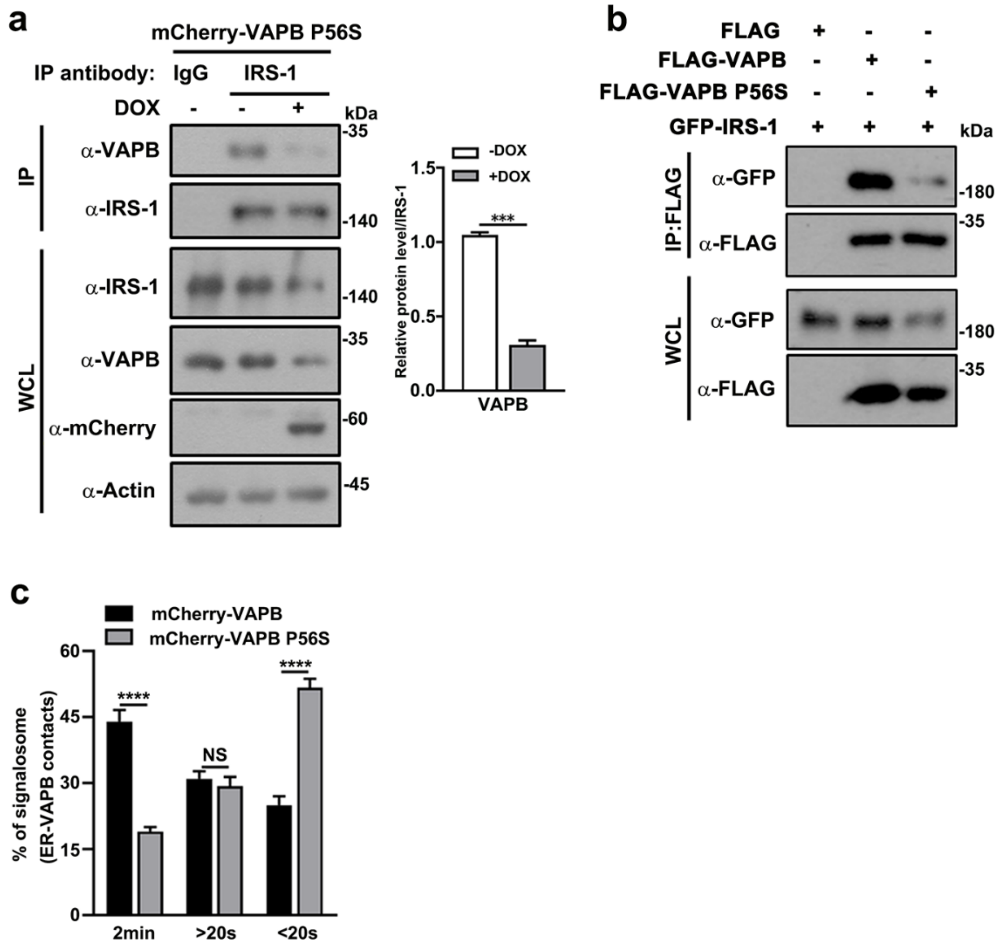

Fig. S9. P56S mutation impairs the interaction between VAPB and IRS-1.

a C2C12 myoblasts stably expressing mCherry-VAPB P56S were subjected to immunoprecipitation with IRS-1 antibodies. Coimmunoprecipitated IRS-1 and VAPB were detected by Western blot analysis. **b** FLAG-tagged VAPB or P56S mutant was co-transfected with GFP-IRS-1 into 293T cells for co-immunoprecipitation analysis. **c** Quantification of association between GFP-IRS-1 droplets and mCherry-VAPB or mCherry-VAPB P56S mutant from 3 biologically independent samples (12 cells for each sample). n=112, 125, or 135 puncta from mCherry-VAPB transfected cells and n=105, 110, or 124 puncta from mCherry-VAPB P56S mutant transfected cells. ****: p <0.0001. NS: not significant.
